# Multiple Peptidoglycan Modification Networks Modulate *Helicobacter pylori's* Cell Shape, Motility, and Colonization Potential

**DOI:** 10.1371/journal.ppat.1002603

**Published:** 2012-03-22

**Authors:** Laura K. Sycuro, Timna J. Wyckoff, Jacob Biboy, Petra Born, Zachary Pincus, Waldemar Vollmer, Nina R. Salama

**Affiliations:** 1 Molecular and Cellular Biology Graduate Program, Fred Hutchinson Cancer Research Center, Seattle, Washington, United States of America; 2 Division of Human Biology, Fred Hutchinson Cancer Research Center, Seattle, Washington, United States of America; 3 Division of Science and Mathematics, University of Minnesota, Morris, Minnesota, United States of America; 4 Centre for Bacterial Cell Biology, Institute for Cell and Molecular Biosciences, Newcastle University, Newcastle upon Tyne, United Kingdom; 5 Department of Molecular, Cellular and Developmental Biology, Yale University, New Haven, Connecticut, United States of America; University of Illinois, United States of America

## Abstract

Helical cell shape of the gastric pathogen *Helicobacter pylori* has been suggested to promote virulence through viscosity-dependent enhancement of swimming velocity. However, *H. pylori csd1* mutants, which are curved but lack helical twist, show normal velocity in viscous polymer solutions and the reason for their deficiency in stomach colonization has remained unclear. Characterization of new rod shaped mutants identified Csd4, a DL-carboxypeptidase of peptidoglycan (PG) tripeptide monomers and Csd5, a putative scaffolding protein. Morphological and biochemical studies indicated Csd4 tripeptide cleavage and Csd1 crosslinking relaxation modify the PG sacculus through independent networks that coordinately generate helical shape. *csd4* mutants show attenuation of stomach colonization, but no change in proinflammatory cytokine induction, despite four-fold higher levels of Nod1-agonist tripeptides in the PG sacculus. Motility analysis of similarly shaped mutants bearing distinct alterations in PG modifications revealed deficits associated with shape, but only in gel-like media and not viscous solutions. As gastric mucus displays viscoelastic gel-like properties, our results suggest enhanced penetration of the mucus barrier underlies the fitness advantage conferred by *H. pylori's* characteristic shape.

## Author Summary

The only habitat of *Helicobacter pylori* is the human stomach, where it can promote stomach ulcers and cancer. Cells lining the stomach are protected from luminal acid by a thick layer of gastric mucus composed of polymerized gastric mucins. Gastric mucin undergoes a physical transition between a viscoelastic solution at neutral pH to a viscoelastic gel-like state at low pH. Helical rod shape in bacteria has been suggested to enhance swimming velocity in viscous solutions by a cork-screw mechanism, but *H. pylori* mutants lacking helical twist show normal swimming velocity in viscous polymer solutions used in prior studies comparing motility across bacterial species. These same mutants, however, show diminished colonization suggesting helical shape promotes stomach infection by another mechanism. Here we identified Csd4, a protease of cell wall tripeptides, which induces curvature in the cell body independently from the changes in cell wall crosslinking previously shown to promote helical twist. Cells lacking Csd4 form straight rods that also show colonization defects but normal velocity in several viscous polymer solutions. Upon examination of motility in gel-like media, however, we discovered that elimination or exaggeration of cell curvature perturbs motility. Thus *H. pylori's* helical shape may aid penetration of gel-like stomach mucus.

## Introduction

*Helicobacter pylori* is a helical rod shaped Gram(-) Proteobacterium with only one known niche, the viscous epithelial mucus layer of the human stomach [1]. Infection with *H. pylori* generally occurs during infancy or childhood, persists through adulthood unless treated, and leads to serious clinical pathologies including peptic ulcer and gastric cancer in 10–20% of those infected [2]. Pathologic examination of gastric biopsy specimens reveals *H. pylori* dispersed within the gastric mucus layer and in direct contact with the gastric epithelial cells [3]. It is believed the bacteria localize to these areas to escape the low pH of the stomach lumen, which they can survive only for a matter of minutes [4], and to avoid elimination by peristalsis.

*H. pylori* requires flagella-mediated and chemosensory-directed motility to access and maintain itself in the mucus layer [5]–[8]. *H. pylori's* helical cell shape may contribute to this process by enabling the bacteria to bore into the mucus layer via a cork-screwing mechanism [9]. More specifically, the turning helical cell body is thought to interact with large polymers to generate torque that enhances translational movement and reduces circumferential slip [10]. Mathematical modeling has predicted helical shape improves propulsion efficiency in the form of speed in viscous polymer solutions [11]. *H. pylori* and *Campylobacter jejuni* have been shown to swim faster at higher viscosities than certain rod-shaped species (e.g. *Escherichia coli*) in solutions of methylcellulose [3], [12].

The cell envelope-embedded peptidoglycan (PG) layer is essential to maintain osmotic stability and cell shape in most bacteria including *H. pylori* [13], [14]. Gram(-) bacteria have a thin layer of PG meshwork in their periplasm [15]. This PG sacculus consists of glycan chains of repeating N-acetylglucosamine-N-acetylmuramic acid (GlcNAc-MurNAc) units that are crosslinked by short peptides attached to MurNAc. During enlargement of the PG sacculus, the disaccharide-pentapeptide precursor lipid II is polymerized and inserted into the sacculus by the coordinated action of PG synthases and hydrolases [16]. Penicillin binding protein 1 (PBP1) is the only PG synthase in *H. pylori* and is predicted to serve as both glycan-polymerizing glycosyltransferase and peptide-crosslinking DD-transpeptidase [17]. The other two high molecular weight PBPs encoded by *H. pylori*, PBP2 and PBP3, are both predicted to act as monofunctional DD-transpeptidases.

Pentapeptides that do not participate in crosslinking can be trimmed by DD-, LD-, and DL-carboxypeptidases (CPases) that successively trim pentapeptides to tetra-, tri-, and dipeptides, respectively. No low molecular weight PBP homologues have been identified in the *H. pylori* genome, but the existence of trimmed peptides in the PG sacculus suggests the existence of these peptidase activities [18]. PG derived tripeptide is an agonist for the intracellular pathogen-associated molecular pattern recognition molecule Nod1 which becomes activated during engagement of the *H. pylori* Cag type IV secretion system. Thus increased tripeptide content of the sacculus could potentially increase proinflammatory activity during *H. pylori* infection [19]–[21].

Bacteria also possess DD-endopeptidases (EPases) for cleavage of peptide crosslinks. We previously identified three genes, *csd1-3*, that encode putative DD-EPases and contribute to *H. pylori's* helical cell shape through alterations in PG crosslinking [13]. Upon deletion of each of these genes individually or in tandem, *H. pylori* assumes various curved rod morphologies. Overexpression of *csd3* (*hdpA*) also alters normal helical shape [22]. Here we identify two additional genes, *csd4* and *csd5*, that promote helical cell shape. One, *csd4*, encodes a zinc metallopeptidase that functions as a CPase on PG tripeptide. *H. pylori* loses nearly all curvature in its absence yielding a straight rod. Csd4 proteins are found throughout the Epsilonproteobacteria and the *Campylobacter jejuni* homologue Pgp1 also promotes helical cell shape [23]. Genetic analyses of cell shape and cell wall composition suggest distinct peptidoglycan modifications cooperatively produce helical morphology. We demonstrate straight rod mutants of *H. pylori* are attenuated in stomach colonization without apparent changes in proinflammatory activity. Finally, in our motility analyses of straight, curved and helical rod shaped *H. pylori* strains, we genetically uncouple specific cell wall modifications from shape phenotypes to identify a role for normal helical shape in directional motility through gel-like media.

## Results

### Loss of helical cell shape results from the disruption of two genes, *csd4* and *csd5*

**Figure 1:**
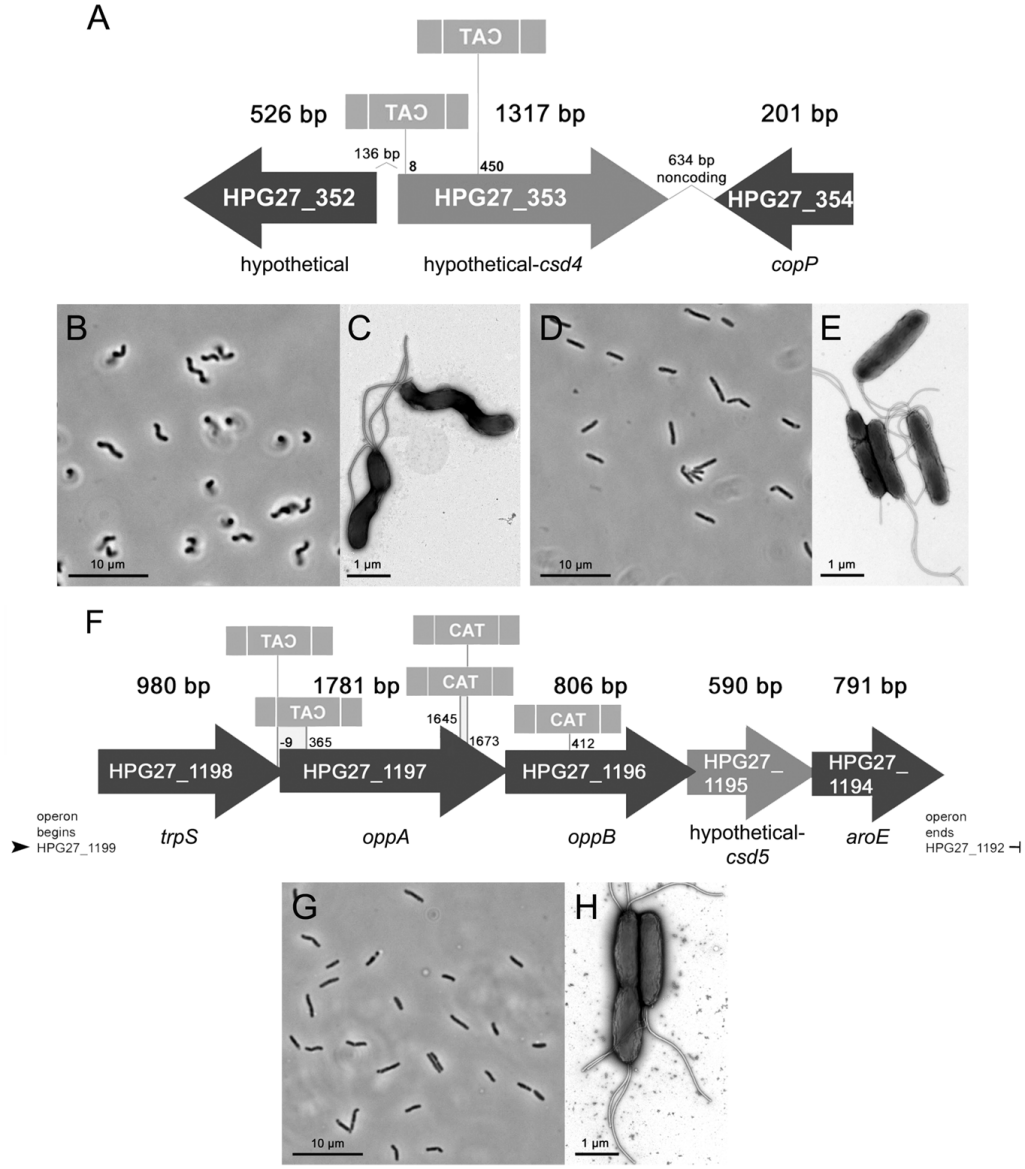
*H. pylori* cell shape mutant morphologies and associated loci identified in a visual screen. The transposon insertion site and orientation (indicated by the spelling of the transposon's selectable marker, chloramphenicol acetyltransferase (*cat*)), is shown for each straight rod shape mutant identified in the screen. A) HPG27_353 (*csd4*) shape locus. B–E) Phase contrast (B, D) and transmission electron microscopy (TEM) (C, E) images of wild-type (B–C) and *csd4* mutant cells (D–E). F) HPG27_1195 (*csd5*) shape locus. G–H) Phase contrast (G) and TEM (H) images of *csd5* mutant cells. Strains used: NSH57, LSH18, LSH31, LSH36.

As previously reported, we discovered the cell shape determinant Csd1, a LytM EPase homologue, in a visual screen of an *H. pylori* transposon mutant library [13]. While the *csd1* mutant has curved rod morphology, two additional mutants with straight rod morphology were also identified in this screen of 2000 random clones. Both transposon insertion sites mapped to HPG27_353 (Figure 1A), a gene encoding a hypothetical protein conserved in *Helicobacter* and other select species in the Delta/Epsilonproteobacteria, all of which are curved or helical (Figure S1A in Text S1). Targeted deletion of HPG27_353 reproduced the rod shape of the transposon mutants (Figure 1D–E) and was complemented by re-expression from the *rdxA* locus (Figure S1C–D in Text S1). Having confirmed HPG27_353 is required for helical curvature and twist in *H. pylori*, we designated this gene *csd4*.

We identified five other transposon mutant clones that display only slightly helical morphology easily distinguishable from wild-type. Each of these mutants contained an insertion in one of two neighboring genes, HPG27_1197 or HPG27_1196, encoding the OppA/OppB members of the oligopeptide ABC transporter that transports small peptides, including PG recycling products, into the cell (Figure 1F) [24]. However, as deletion of each of these genes resulted in cells with normal helical morphology (data not shown), we suspected the transposons affect another gene in the operon. Upon deleting the gene immediately downstream, HPG27_1195, we obtained cells with largely straight rod morphology, though unlike *csd4* mutants, some cells have slight irregular bends and curves (Figure 1G–H). Helical cell shape was restored with complementation (Figure S1C–D in Text S1). HPG27_1195 encodes a hypothetical protein well-conserved in *H. pylori* and the closely related species *H. acinonychis*, but not other Epsilonproteobacteria (Figure S1B in Text S1). We named this gene *csd5*.

### *csd4* and *csd5* mutants grow normally and show minimal alterations in cell length and width

Despite their dramatically altered morphology, *csd4* and *csd5* mutants grew as well as wild-type through log and into stationary phase in broth culture (Figure S2A in Text S1). Neither mutant showed growth deficiency in 72 hrs of log-phase co-culture with wild-type (Figure S2B–C in Text S1). Aside from loss of helical rod shape, neither mutant had any other deformity; formation of cell poles and division septa appeared normal for both mutants, as did polar flagellation (Figure 1E, H, and data not shown). Each mutant is slightly longer than wild-type and the *csd5* mutant is also slightly wider than wild-type, but these differences represent changes of less than 10% (mean length/width in microns: wild-type 2.39/0.58; *csd4*: 2.62/0.58; *csd5*: 2.62/0.62). Both mutant strains underwent coccoid transformation in late stationary phase with similar kinetics to wild-type, showing 100% transformation at 72 hrs (data not shown).

### Csd4 exhibits DL-carboxypeptidase activity required for its shape-determining function

**Figure 2:**
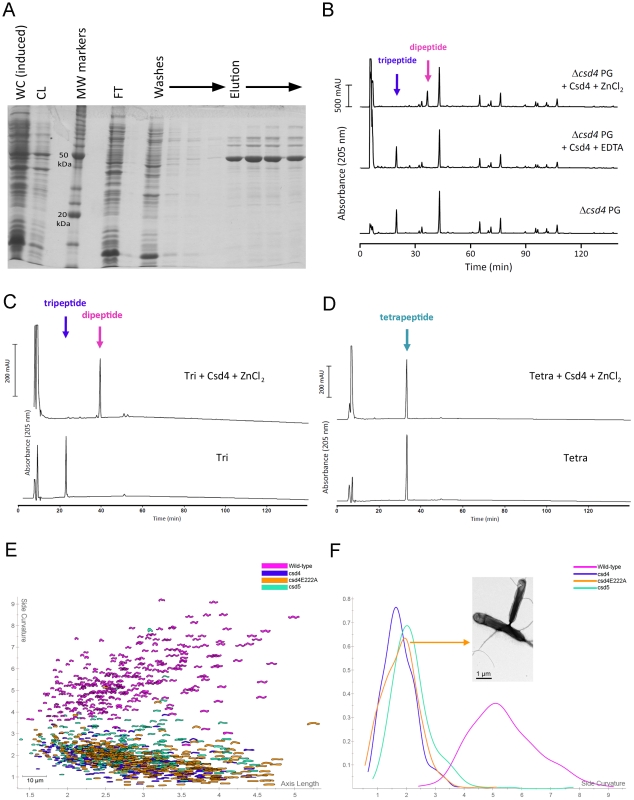
Functional analyses of Csd4 enzymatic activity and its role in shape determination. A) SDS-PAGE depicting steps in the purification of His-tagged *H. pylori* Csd4 protein from *E. coli* cells. Induced protein was purified using a Ni-NTA agarose column as described in Text S1. WC, whole cell lysate; CL, cleared lysate; MW, molecular weight; FT, flow through. Positions of the 20 kDa and 50 kDa molecular weight markers are indicated. B) HPLC analysis of muropeptides released from purified *csd4* mutant (LSH122) PG treated with purified His-tagged Csd4 protein in the presence of Zn^2+^ or EDTA, or without protein. In the presence of Zn^2+^ but not EDTA, Csd4 trimmed the monomeric tripeptides to dipeptides, indicative of the protein having DL-carboxypeptidase activity. C–D) Muropeptides detected before and after incubation of Csd4 with purified disaccharide tripeptide (C) and disaccharide tetrapeptide substrates (D). Data indicate Csd4 cleaves tripeptide, but not tetrapeptide. E–F) Scatter plot arraying the wild-type, *csd4* deletion, *csd4* point, and *csd5* deletion mutant populations by length (x-axis, µm) and cell curvature (y-axis, arbitrary units). Each contour depicts the morphology of a single cell captured from a 1000× phase contrast image using CellTool software [13]. The software algorithmically determines each cell's length along its two-dimensional central axis as well as the degree of cell body curvature (excluding the poles). 200–300 cells were analyzed for each strain. E) Smooth histograms displaying kernel density estimates of each strain's cell curvature (x-axis). Bootstrapped Kolmogorov–Smirnov statistical comparisons of population cell curvature distributions yielded p-values<0.001 for all pairwise comparisons with the exception of *csd4* vs. *csd4E222A*, p = 0.19. Strains used: NSH57, LSH18, LSH31, LSH146.

Csd4 contains a putative N-terminal signal sequence and an M14 peptidase domain, the latter placing it in the zinc-dependent carboxypeptidase superfamily [25]. One of the few well-characterized bacterial M14 peptidases is *Bacillus sphaericus* endopeptidase I, which cleaves the D-glutamic acid-*meso*-diaminopimelic acid (D-Glu-mDap) peptide linkage of PG tetrapeptides (EPase activity) and tripeptides (CPase activity) [26], [27]. Due to its involvement in cell shape determination, we hypothesized Csd4 may also exhibit endo- or carboxypeptidase activity on PG substrates. We over expressed His-tagged Csd4 protein in *E. coli* (Figure 2A) and tested enzymatic activity of the purified protein *in vitro* using sacculi from a *csd4* mutant strain as substrate. In the presence of Zn^2+^, Csd4 removed virtually all monomeric (uncrosslinked) tripeptides, yielding dipeptides (Figure 2B). No reaction was observed in the buffer control or when the enzyme sample contained EDTA, confirming its dependency on divalent cations such as Zn^2+^. No other muropeptide species showed significant change (Table S1 in Text S1), indicating Csd4 is a DL-CPase that trims monomeric tripeptides to dipeptides. Further confirmation of Csd4's substrate specificity was obtained using purified disaccharide tripeptide and disaccharide tetrapeptide monomers; Csd4 was enzymatically active against the tripeptide, but not the tetrapeptide species (Figure 2C–D).

Structural threading of the Csd4 protein sequence revealed several strong matches to M14 peptidases with solved crystal structures, including human carboxypeptidase M (*E*-value 1.8E^−6^) [28], [29]. Positional mapping of residues involved in zinc binding and catalysis on the carboxypeptidase M crystal structure enabled the deduction of corresponding residues on the threaded Csd4 structure (Figure S3 in Text S1). We identified Csd4 E222 as a candidate for the catalytic glutamate and targeted this residue using site-directed mutagenesis. A single nucleotide substitution in the *csd4* gene, A665C, resulted in an E222A substitution in the protein. Allelic exchange was used to introduce the mutant allele at the endogenous locus [30]. The *csd4E222A* mutant strain had straight rod morphology (Figure 2E–F), suggesting Csd4 CPase activity is vital for generating helical cell shape.

### PG of rod-shaped mutants is altered in the abundance of monomeric muropeptides and in peptide crosslinking

We sought further evidence of Csd4 DL-CPase activity *in vivo* by comparing the PG sacculus muropeptide composition of the *csd4* and *csd4E222A* mutants to wild-type and a complemented strain (Table 1 and Table S2 in Text S1). The point mutant and null mutant strains showed identical muropeptide profiles. The most striking differences in the PG of both mutants was a >400% increase in monomeric tripeptide and the absence of virtually all monomeric dipeptide (Table 1), suggesting Csd4 catalyzes the trimming of tripeptides to dipeptides via its DL-CPase activity, as we observed in vitro. Both *csd4* mutants also showed changes in other muropeptides, most notably a >400% decrease in tetrapeptide and increases and decreases in various crosslinked species. *csd4* mutants showed an approximately 300% increase in tetra–tripeptide crosslinking while tetra–tetrapeptide and tetra–pentapeptide crosslinked dimers were both reduced (by 51% and 12%, respectively). Since tetra–tripeptide crosslinks are not very abundant in the wild-type cells and the other more abundant crosslinked species were decreased, the overall degree of crosslinking was unchanged in the mutants.

**Table 1:** Summary of muropeptide composition of PG in mutant strains.

	Area - % of Each Muropeptide^a^										
	Wild-Type (Avg ± SD)^b^	*csd1* c	*csd3* c	*csd4*	*csd4^E222A^*	*csd5*	*csd4 csd5*	*csd1 csd4*	*csd1 csd5*	*csd3 csd4*	*csd3 csd5*
	h^d^	c^d^	v^d^	s^d^	s	s	s	c	c	c	s
Monomers (total)	58.7±1.7	54.7	54.8	60.9	60.0	59.3	57.6	55.3	53.2	59.6	56.2
Dipeptide	2.8±0.4	1.7	2.3	**0.0**	**0.0**	2.8	**0.0**	**0.4**	1.9	**0.0**	2.5
Tripeptide	4.0±0.4	4.7	3.6	**17.3**	**16.1**	4.5	**17.8**	**12.8**	3.4	**11.7**	3.6
Tetrapeptide	10.0±0.6	7.4	**6.8**	**2.3**	**2.1**	8.9	**1.4**	**2.3**	7.5	**3.2**	**7.0**
Pentapeptide	41.8±1.1	40.9	42.0	41.3	41.8	43.2	38.4	39.8	40.5	44.7	43.2
Dimers (total)	41.3±1.7	45.3	45.2	39.1	40.0	40.7	42.4	44.7	46.8	40.4	43.8
Tetra–tri	4.5±0.3	4.0	3.4	**12.8**	**13.2**	4.7	**14.5**	**9.3**	3.7	**7.5**	3.3
Tetra–tetra	15.8±0.3	14.5	**9.9**	**7.8**	**7.5**	14.3	**7.8**	**9.6**	15.5	**5.7**	**8.4**
Tetra–penta	21.1±1.3	26.9	**31.8**	18.5	19.3	21.7	20.1	**25.8**	27.6	27.2	**32.1**
Chain ends (anh)	10.3±0.6	8.4	9.5	8.6	9.0	9.3	10.5	9.2	10.4	9.1	8.9
Avg Chain Length	9.7±0.6	12.0	10.6	11.7	11.1	10.8	9.6	10.9	9.6	11.0	11.3
% Peptides in Crosslinks	41.3±1.7	45.3	45.2	39.1	40.0	40.7	42.4	42.5	44.7	46.8	43.7

a Percentages calculated as per [54]. Underlined values differ by more than 2 standard deviations from that of wild-type; underlined and bold values differ by more than 5 standard deviations from that of wild-type.

b Calculated from 6 independent samples.

c As previously reported [13].

d Shape of each strain: h-helical rod, c-curved rod, s-straight rod, v-variable (“c” shape, curved rod, straight rod).

We found a markedly different PG profile for the *csd5* mutant compared to the morphologically similar *csd4* mutants (Table 1). The *csd5* mutant exhibited very modest increases in tetra–tripeptide crosslinks and monomeric tripeptides compared to wild-type (increased by 4% and 13%, respectively). The *csd5* mutant strain also showed modest decreases in tetra–tetrapeptide crosslinks (9%) and monomeric tetrapeptides (11%). Similar decreases in tetrapeptide-containing species occurred in the LytM homologue mutants (*csd1, csd3*), as well as *csd4* mutants (Table 1). Csd5 does not contain any known enzymatic domains, but does contain a bacterial SH3 motif, which could play a role in protein-protein interactions or PG binding [31]–[33]. A *csd4csd5* double mutant strain has a straight rod shape and displays a more severe loss of curvature than the *csd5* mutant and overlaps the *csd4* mutant profile (Figure S4A–B in Text S1). Furthermore, the global PG profile of the *csd4csd5* strain mirrors that of *csd4* mutants (Table 1). Altogether these findings indicate that perturbation of monomeric and/or crosslinked PG species influences *H. pylori* cell shape, but global cell wall perturbations are not necessary for loss of helical cell shape, as the *csd5* mutant PG profile remains similar to wild-type.

### Genetic interactions among *csd* genes reveal two distinct PG modification networks that promote helical cell shape

**Figure 3:**
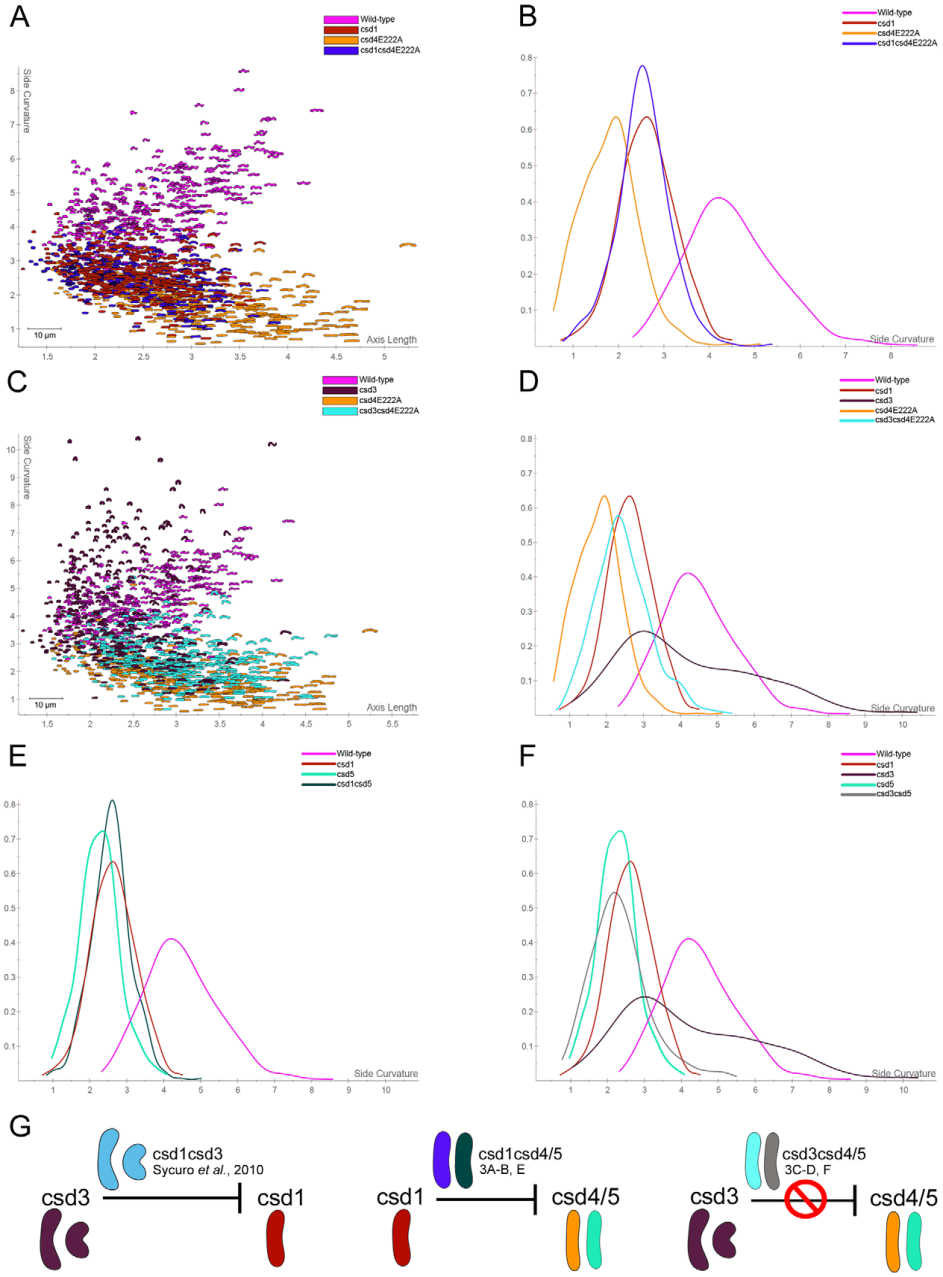
Morphological characterization of single and cross-shape class double mutants. A, C) Scatter plots arraying wild-type and mutant populations by cell length (x-axis, µm) and cell curvature (y-axis, arbitrary units). B, D–F) Smooth histograms displaying population cell curvature (x-axis) as a density function (y-axis). Bootstrapped Kolmogorov–Smirnov statistics of population cell curvature distributions: B) *csd1csd4* vs. *csd1* p = 0.31, *csd1csd4* vs. *csd4* p<0.00001; D) *csd3csd4* vs. *csd4* p<0.00001, *csd3csd4* vs. *csd3* p<0.00001, *csd3csd4* vs. *csd1* = 0.0055; E) *csd1csd5* vs. *csd1* p = 0.25, *csd1csd5* vs. *csd5* p<0.00001; F) *csd3csd5* vs. *csd5* p = 0.37, *csd3csd5* vs. *csd3* p<0.00001, *csd3csd5* vs. *csd1* p<0.00001. All mutant comparisons to wild-type had p<0.00001 (B, D–F). G) Summary of morphological epistasis relationships. The average cell contour model generated by CellTool is shown for each strain. Two contours are shown for the morphologically variable *csd3* mutant representing two distinct aspects of the shape distribution of this mutant (curvature values of 3 (mode) and 6 (right-hand tail)). Strains used: LSH100, LSH123, LSH134, LSH146, MLH3, MLH4, NSH152a, NSH153a, NSH160a, NSH161.

Our earlier work revealed that mutation of *csd1*, *csd2*, or *ccmA* individually or in combination results in curved rod morphology and increased tetra–pentapeptide crosslinking [13]. We employed genetic interaction studies to determine the relationship between the *csd1* network and *csd4* and *csd5*. We found that *csd1csd4* and *csd1csd5* double mutants are both curved like the *csd1* mutant (Figure 3A, B, E, S4C in Text S1). Both double mutants also accumulated excess tetra–pentapeptide crosslinks in the PG sacculus similar to *csd1* (Table 1).

Csd3 is a predicted homologue of Csd1 and Csd2, but *csd3* mutants show a distinct cell shape profile comprised of specific ratios of straight rods, curved rods, and highly curved “c” shapes [13]. Combined mutation of *csd3* along with *csd1csd2ccmA* gave rise to a population morphologically indistinguishable from the *csd3* mutant, indicating *csd3* is epistatic to and perhaps upstream of these other shape-generating genes [13]. However, the *csd3* cell shape phenotype was not epistatic to *csd4* or *csd5*. The *csd3csd4* mutant displayed a curved rod shape distinct from *csd3* and the straight rod phenotype of *csd4* (Figure 3C–D), while the *csd3csd5* mutant retained a side curvature profile very similar to that of *csd5* (Figure 3F, S4D in Text S1). PG analysis of these double mutants again showed increases in tetra–pentapeptide crosslinked dimers (Table 1).

In summary, the shape phenotypes of double mutants of *csd1* and *csd3* with either *csd4* or *csd5* showed evidence of epistasis (Figure 3G), whereas their PG profiles were largely additive (Table 1). For example, both Csd1-dependent increases in tetra–pentapeptide crosslinked species and Csd4-dependent increases in tripeptide monomer were present in the *csd1csd4* mutant. As the exception, tetra–tripeptide crosslinking was increased in the *csd4* mutant, decreased in the *csd3* mutant, and at an intermediate level in the double mutant. Together these findings suggest that Csd4 DL-CPase activity does not depend on LytM EPase activity and vice versa. However, Csd3 and Csd4 have opposing influences on the abundance of tetra–tripeptide crosslinks in the sacculus.

### Straight rod mutants show impaired stomach colonization but no disruption of cell wall integrity

**Figure 4:**
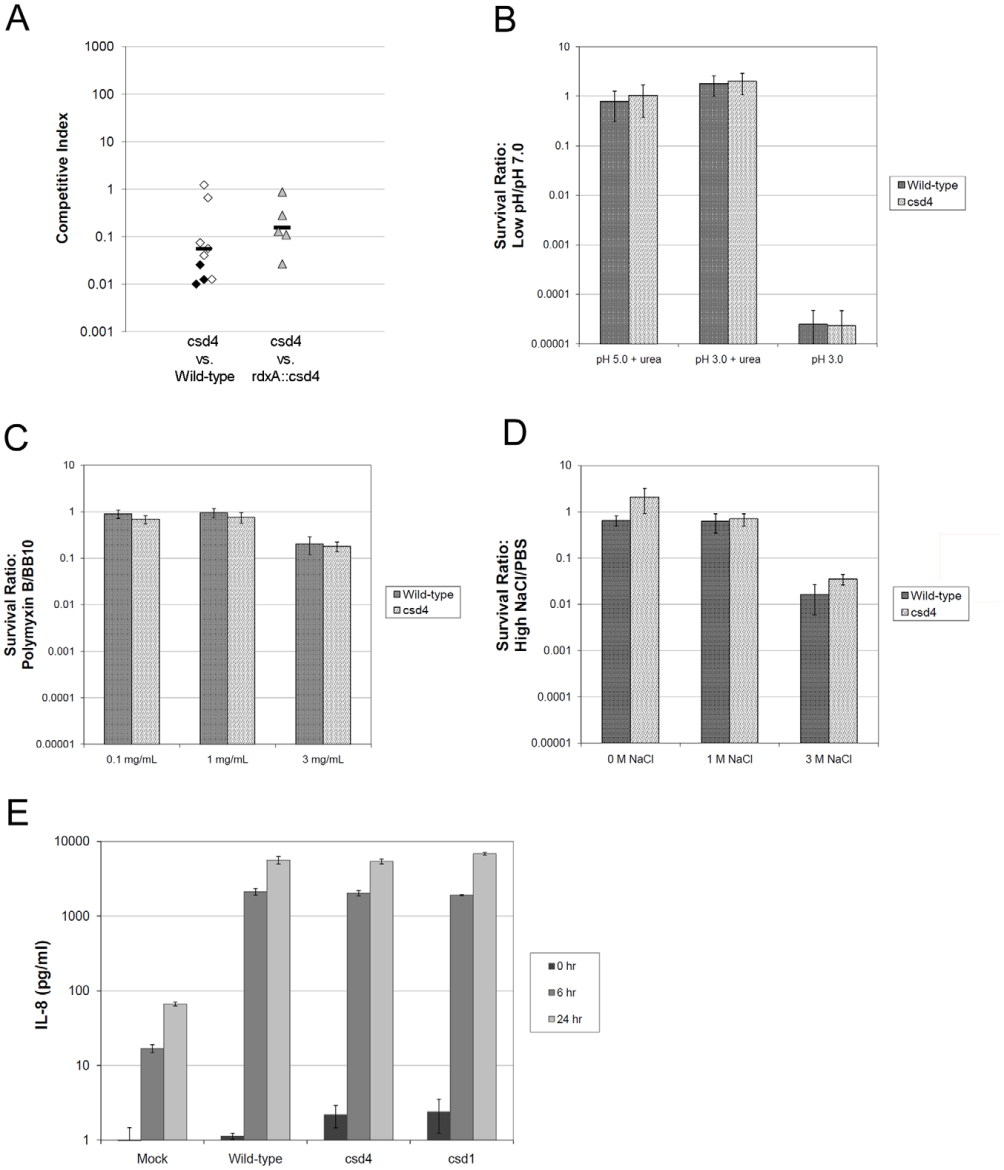
Assessment of the straight rod *H. pylori*'s colonization and pro-inflammatory potential. A) One week C57BL/6 mouse competition data compiled from three independent experiments. Data are plotted as a competitive index: [CFU/mL_MUT_∶CFU/mL_WT/Complement_ stomach output]/[CFU/mL_MUT_∶CFU/mL_WT/Complement_ inoculum] with each data point representing a single mouse. Black points indicate mice from which only one strain was recovered. Strains used: LSH100, LSH122, LSH124. B–D) Survival at low pH (B), in the presence of polymyxin B (C), or in high salt (D). Data comprise two independent experiments of four replicates per strain and condition (mean ± SD). Strains used: NSH57, LSH18. E) IL-8 production during infection of AGS gastric epithelial cells. Culture supernatants of triplicate wells were assayed for IL-8 using a commercial ELISA assay after infection at a multiplicity of infection of 10 (mean ± SD). Shown are data from one of three independent experiments with similar results. Strains used: NSH57, LSH13, LSH18.

Previous work revealed *csd1* curved rod mutants and *csd3* variably curved rod mutants are attenuated in stomach colonization [13], [22]. As *csd4* mutants are the straightest of the two rod-shaped mutants, we focused further characterization on this mutant to understand the impact of its dramatic cell shape change on stomach colonization. The *csd4* mutant was strongly outcompeted by wild-type and the *csd4* complemented strain in the C57BL/6 mouse model (Figure 4A). In contrast, during co-culture in broth no competitive defect was observed (Figure S2B in Text S1), suggesting the cell shape and/or cell wall changes present in this mutant are uniquely required during stomach colonization.

PG is both a stress-bearing structure responsible for withstanding turgor pressure and a dynamic part of the assembly and function of many cell wall protein complexes. We thus tested whether alterations in its chemical content might alter the function of the wall so as to render the cells less able to survive environmental stresses *H. pylori* encounters in the stomach: acid, antimicrobial peptides, and osmotic stress. The *csd4* mutant survived exposure to low pH, an antimicrobial peptide similar to those found in the stomach (polymyxin), and high salt as well as wild-type (Figure 4B–D). These results show that the cell wall changes produced by the loss of *csd4* do not appreciably alter cell wall integrity and further support a direct role for normal shape in efficient stomach colonization.

### Elevated PG tripeptide content or crosslinking of the PG sacculus does not alter innate immune detection of live *H. pylori*

Successful stomach colonization by *H. pylori* requires penetration of the gastric mucus and intimate association with the epithelium. Once contact with the host epithelium is established, the Cag type IV secretion system (T4SS) engages host cells and exposes them to toxins that are associated with more serious disease outcomes [2]. The Cag T4SS induces pro-inflammatory cytokine secretion by introducing PG fragments into the host cell, which activates the mammalian intracytoplasmic pathogen recognition molecule Nod1 and ultimately NFκB [20], [34], [35]. All our cell shape mutants show changes in global PG composition and several have increased overall crosslinking of the cell wall (Table 1). Of particular interest, the *csd4* mutants showed profound accumulation of mDap-containing tripeptide monomers, which are Nod1 agonists [19], [21], [36]. The wild-type strains used in our studies contain the *cag* pathogenicity island (PAI) that encodes the Cag T4SS and thus induce robust secretion of IL-8 upon co-culture with the AGS gastric epithelial cell line [37], [38]. We wondered whether the increased crosslinking of the *csd1* mutant sacculus interferes with periplasmic assembly of the Cag T4SS or if the *csd4* mutant would elicit higher IL-8 induction due to the higher tripeptide content of the sacculus. As shown in Figure 4E, neither mutant showed increased or decreased IL-8 secretion relative to wild-type. Thus altered PG crosslinking in several cell shape mutants likely does not impair Cag T4SS assembly and the extra tripeptide in the *csd4* mutant PG sacculus may not be available for host cell delivery by the Cag T4SS.

### Shape-dependent motility phenotypes are confined to gel-like media

**Figure 5:**
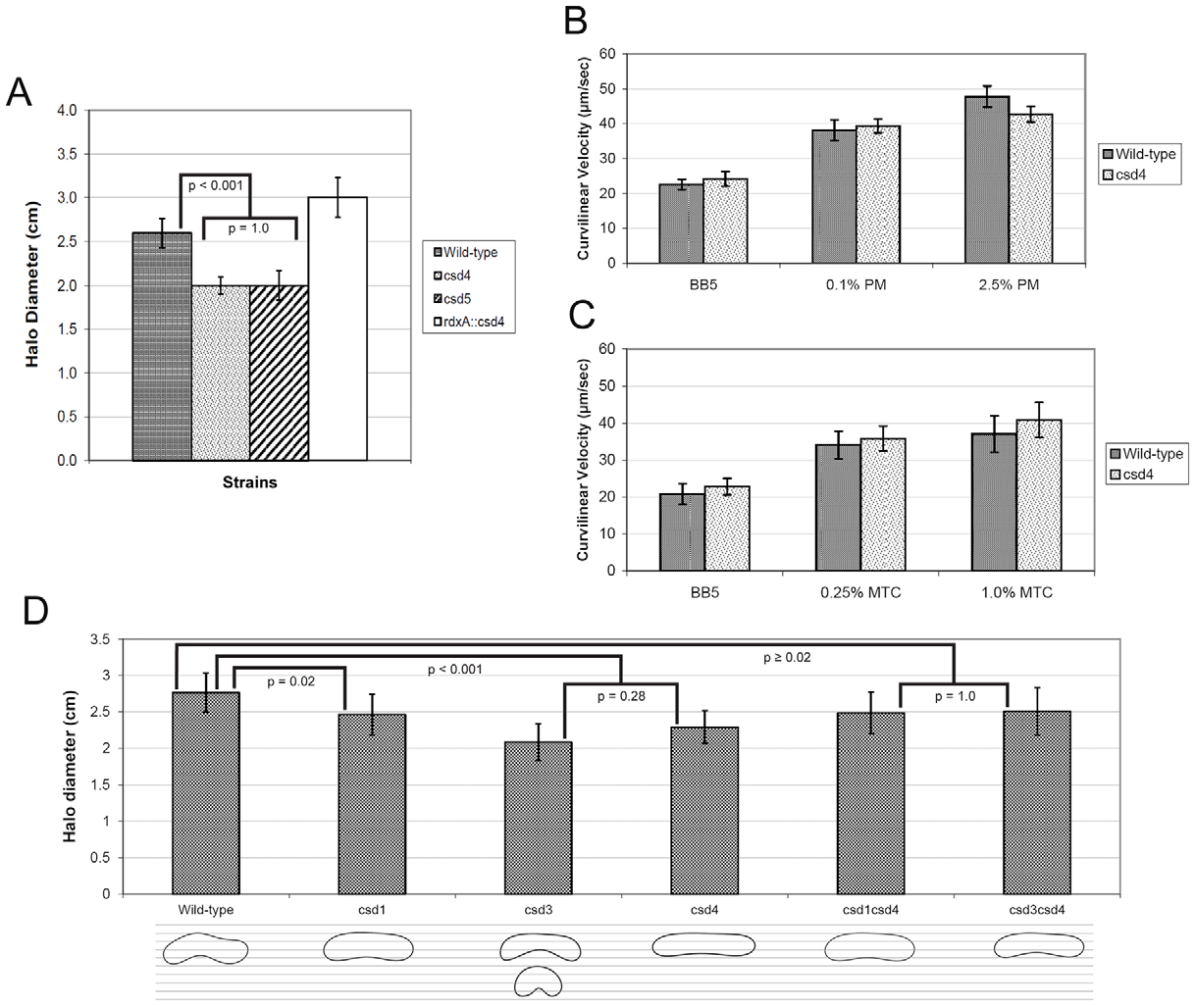
Motility of *H. pylori* cell shape mutants in soft agar and viscous polymer solutions. A, D) Motility phenotype of indicated strains in soft agar (mean halo diameter ± SD in 0.3% soft agar after four days). Data shown are from one experiment of 17–22 stabs/strain and are consistent with the findings from replicate experiments. Contours representative of each strain's average cell shape (see Figure 3 legend) are shown below panel D and are superimposed on a grid to highlight the slight differences in cell curvature that correlate with motility. p-values were generated using one-way ANOVA with the Bonferroni correction for multiple comparisons. B–C) Velocity of wild-type and the *csd4* mutant in broth containing porcine mucus (B) and methylcellulose (C). Data shown are the mean ± SD from measurements of 9–30 cells/strain/condition. No statistically significant differences between wild-type and the *csd4* mutant were observed in any condition (p>0.2, Student's t-test with equal variances). Strains used: A) LSH100, LSH122, LSH123; B–C) NSH57, LSH18; D) LSH100, LSH134, NSH152a, LSH146, NSH153a, NSH160a.

As neither cell wall integrity nor innate immune detection appear to explain the colonization defects of the *csd4* mutant we investigated motility. The *csd4* and *csd5* mutants were highly motile in broth culture, but were deficient in a soft agar motility assay, generating halos that were ∼20% smaller than wild-type on day four (Figure 5A, D). The mutant phenotype of *csd4* was reversed by reintroduction of the gene at a distal locus. Motility in soft agar depends on many aspects of swimming behavior including velocity, switching of flagellar rotation in response to chemosensory cues, and ability to bore through the pores of the gel. The cork-screw premise predicts helical-shaped cells will swim more rapidly than rod-shaped cells at high viscosities [9], so we compared the swimming velocity of the *csd4* mutant to wild-type. *csd4* mutants swam at the same velocity as wild-type in broth and in three different viscous polymer solutions: crude porcine mucin, methylcellulose, and Ficoll (Figures 5B–C, Videos S1, S2, and data not shown). However, even the highest polymer concentrations used in this experiment do not mimic the viscoelastic gel-like properties of gastric mucus [39].

We thus returned to our gel-like soft agar assay to further explore the relationship between motility and shape under gel-like conditions. We previously reported that mutants lacking helical twist but retaining curvature (*csd1, csd2, ccmA*) formed halos in soft agar gel similar to wild-type bacteria while the variably shaped *csd3* mutant was significantly deficient in soft agar halo formation [13]. As shown in Figure 5D, *csd1* mutants made halos 11% smaller than wild-type (p = 0.02), whereas both *csd3* and *csd4* mutants show more significant reductions in halo size (25% and 17%, p<0.001). We then examined the motility phenotype of the *csd3csd4* double mutant, which is morphologically similar to the *csd1* mutant (Figure 3D), but has a significantly different PG profile (Table 1). The *csd3csd4* double mutant's motility in soft agar is similar to the *csd1* mutant (9% reduction compared to wild-type, p = 0.05, Figure 5D). The *csd1csd4* mutant (another strain morphologically similar to *csd1*, but with a different PG profile) also showed enhanced motility relative to the *csd4* mutant with a halo formation phenotype indistinguishable from the *csd3csd4* mutant (p = 1.0, Figure 5D). Partial suppression of the soft agar motility phenotypes of the *csd3* and *csd4* mutants suggests a relationship between shape and motility whereby more severe perturbations of shape, including large increases (“c” shape) or decreases (straight rod) of curvature lead to more severe attenuation of directional motility in gel-like media compared to strains that have curvatures profiles closer to those of wild-type (*csd1, csd3csd4, csd1csd4*, Figures 3, 5D).

## Discussion

Our collection of genetically defined and morphologically diverse cell shape mutants enabled us to establish a connection between cell shape and motility in *H. pylori*, but exclusively in gel-like media. *H. pylori* motility in gel-like media decreases with increasing perturbation of cell shape such that the straight rod *csd4* mutant shows greater defects than the curved rod *csd1* mutant. The motility defect of the *csd4* mutant is partially suppressed by *csd3* or *csd1* mutation and since the PG peptide changes in the sacculus were largely preserved in the double mutants, the partial suppression of the motility phenotype correlates most strongly with the reintroduction of cell curvature. We were unable to detect shape-dependent velocity changes in viscous polymer solutions, but future experiments in purified gastric mucin that retains gel-like properties may reveal velocity defects of the straight and/or curved rod mutants. In addition to velocity, altered cell shape may affect chemotaxis, particularly since *H. pylori* does not tumble but relies on Brownian forces for redirection. Shape may also alter the cells' ability to swim straight, as is the case for some of the “c”-shaped cells in the *csd3* mutant population, which swim in circles [13]. Altogether our findings provide evidence that *H. pylori*'s tight control of cell shape is critical for optimal motility in the stomach environment.

While our *in vitro* experiments showed only subtle perturbations of motility, particularly for curved rod shaped mutants, all *H. pylori* mutants with non-helical morphology tested to date (curved rod *csd1*, variably shaped *csd3*, and straight rod *csd4*) are deficient in a mouse colonization assay [13], [22]. We explored whether loss of cell wall integrity might underlie the observed colonization defects, but our mutants do not show increased sensitivity to pH, high osmolarity or the antimicrobial peptide polymyxin. We also investigated the possibility that altered colonization is secondary to changes in innate immune detection of *H. pylori*-derived PG, but found no evidence for alteration of proinflammatory cytokine induction by mutants with increased PG crosslinking or monomeric tripeptides. The *cag* PAI-encoding strain of *H. pylori* used in our infection experiments induces a Nod1-mediated proinflammatory response capable of affecting *Helicobacter* loads in the mouse [40], [41], but the source of PG fragments delivered to host cells by the Cag T4SS is not clear. In contrast to previous work showing enhanced Nod1 activation when cultured HEK293 cells were treated with digested purified *H. pylori* sacculi containing elevated tripeptide [20], our results suggest that the tripeptide content of the sacculus does not correlate with Nod1 activation in gastric epithelial cells during infection with live bacteria. Efficient directional motility is required for robust stomach colonization [5], [7], [8], [42], suggesting the colonization defect of *csd4* (and *csd1* and *csd3*) mutants relates to altered motility. As we could only measure motility defects in gel-like media and gastric mucin attains gel-like properties only at low pH [39], helical shape may be particularly required for penetration of the more luminal (and acidic) mucus layer of the stomach to gain access to its extracellular niche within the more neutral, cell proximal gastric mucus.

In addition to helical morphology, another defining characteristic of *Helicobacter pylori* is its highly plastic genome. As described here and in a previous study [13], microscopic analysis of 2000 randomly mutagenized clones yielded nine mutants with altered cell shape. This rather limited screen led to the discovery of six genes required for helical cell shape but not cell growth, cell polarity (as measured by normal polar flagellar assembly), or the coccoid cell shape transformation that occurs in late stationary phase. Each of these genes is conserved in all *H. pylori* genomes that have been sequenced to date, suggesting *H. pylori* maintains a complex molecular program dedicated to promoting helical rod shape during log phase growth. In contrast, the recently described *H. pylori* coiled coil rich proteins (Ccrp), which form cytosolic filaments and may influence cell shape, are variably present across strains [43].

**Figure 6:**
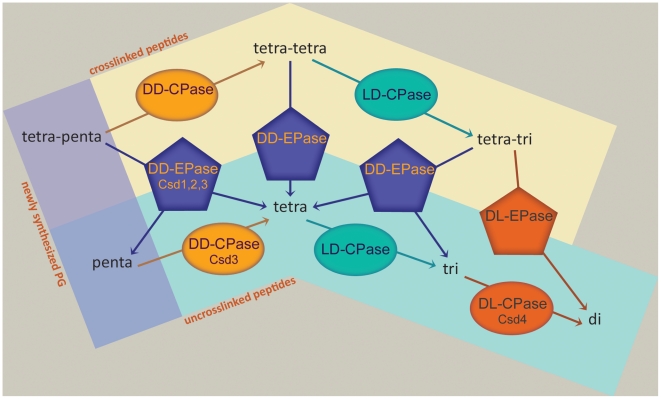
Current understanding of muropeptide modification in *H. pylori*. This schematic shows peptide modification activities that can generate the muropeptides observed in the *H. pylori* sacculus. Known *H. pylori* proteins demonstrated (Csd3, Csd4) or predicted (Csd1, Csd2) to perform these activities are indicated. CPase, carboxypeptidase; EPase, endopeptidase.

Unlike *E. coli*, *H. pylori* contains high levels of uncrosslinked pentapeptide in the PG sacculus [18] and does not encode low molecular weight penicillin binding protein homologues. However, three cell shape-determining genes encode DD-EPases/CPases (Csd1-3) [13], [22], and here we show *csd4* encodes a DL-CPase (Csd4). Thus remodeling of PG peptides does occur in this organism (Figure 6). Our PG analysis and *in vitro* assay of protein activity show that Csd4 has DL-CPase activity on tripeptide monomers, cleaving the terminal mDap residue to produce dipeptide monomers. Additional enzymes must convert uncrosslinked pentapeptides into tetrapeptides and tetrapeptides into the tripeptide substrate of Csd4. Csd3 was shown to have *in vitro* DD-CPase activity on a monomeric pentapeptide substrate in addition to DD-EPase activity on tetra–pentapeptide dimers [22] and thus may initiate a trimming cascade on uncrosslinked muropeptides in *H. pylori*. However, *csd3* PG changes are not epistatic to *csd4*, which suggests Csd3 is not required to generate Csd4 tripeptide substrate and insinuates the existence of another peptidase with redundant DD-CPase activity.

How trimming of uncrosslinked muropeptides by Csd3, Csd4, and likely other proteins contributes to cell shape remains to be determined. *H. pylori* may control the availability of specific monomeric species to limit or localize the formation of crosslinks. We and others have proposed models of cell curvature and twist based on the overall and/or localized extent of PG crosslinking [13], [44]. Since the transpeptidation reaction requires both donor pentapeptides and mDap-containing acceptors (penta-, tetra-, or tripeptides), the dipeptide-generating function of Csd4 may prevent crosslinking in certain regions of the sacculus. As such, the increase in tetra–tripeptide crosslinking observed in *csd4* mutants could simply result from the overabundance of crosslinking-active tripeptide in the sacculus. This scenario seems likely since purified Csd4 only shows activity on monomeric species. Alternatively, the occurrence of shorter monomeric species, namely dipeptides, in the sacculus is thought to signify “old” PG and may serve as a signal for the synthesis machinery to assemble and insert new PG [16], [45]. Localized differences in the rate of PG synthesis have been shown to drive cell curvature in *Caulobacter crescentus* [46].

Surprisingly, the *csd5* mutant shows negligible perturbations of global PG composition indicating Csd5 is not required for Csd4 enzymatic activity. The observations that *csd1* is epistatic to both *csd4* and *csd5*, and that *csd3* is epistatic to neither *csd4* nor *csd5*, suggest that *csd4* and *csd5* act at a similar stage of helical cell shape specification. The *csd4csd5* double mutant resembled the *csd4* mutant both in global PG changes and by having a straighter shape than the *csd5* mutant. Csd5 bears a probable transmembrane domain or signal sequence allowing localization to the inner membrane and/or periplasm, as well as a bacterial SH3 domain in the C-terminus, which could allow for interactions with other PG peptidases and/or PG. The epistasis of *csd4* on *csd5* could suggest Csd5 acts downstream of Csd4. Csd5 might recognize dipeptides generated by Csd4 enzymatic activity and provide an activating signal for cell shape modulation, perhaps through recruitment of PG synthesis enzymes. Alternatively, Csd5 may localize Csd4 activity in a particular pattern conducive for helical cell shape generation. In this model, absence of Csd5 would lead to randomly located Csd4 activity which could alter cell shape without altering the global PG composition. Recently two lipoprotein activators of the major PG synthases in *E. coli* have been identified, providing a paradigm for localized activation of PG modifying enzymes [47], [48].

The PG modifications caused by the straight and curved rod classes of shape genes appear largely independent. There is no epistasis in the PG phenotypes of the double mutants we tested; each double mutant shows changes that are additive or intermediate compared to the single mutant phenotypes. Moreover, we do not observe a genetic hierarchy of shape phenotypes; straight rod shape is not epistatic to the seemingly more complex curved and helical rod shapes. Instead, the curved rod shape of *csd1* is epistatic to the straight rod shape of *csd4* and *csd5*. Our genetic interaction studies suggest a minimum of two distinct networks that alter PG and cell shape in *H. pylori*: a network containing Csd1, Csd2, and CcmA that generates helical twist through relaxation of tetra–pentapeptide crosslinking and a network containing Csd4 and Csd5 that generates curvature through some consequence of monomeric muropeptide trimming. While straight rod *csd4* and *csd5* mutants appear to lack curvature and twist, we cannot be certain whether their protein activities contribute to twist or whether the activities of Csd1, Csd2, and CcmA are generating twist in the absence of Csd4/5, but in a manner that is not apparent in the absence of curvature. Csd3 appears to play a role in both networks, as it has activity on both crosslinked and uncrosslinked muropeptides. Further refinement of a model incorporating these complex modifications of crosslinked and uncrosslinked muropeptide species in the generation of helical cell shape will require further characterization of Csd and CcmA protein activities and spatial organization, as well as identification of missing peptidases and other co-factors.

Some components of the *H. pylori* helical shape-generating program are found throughout the Proteobacteria while others appear subdivision- or even species-specific. Homologues of the LytM peptidase Csd1 are the most widely conserved and found in all subdivisions of the Proteobacteria, but not exclusively in organisms with curved to helical shape [13]. Several species have more than one Csd1 homologue (up to 9), including *H. pylori* (Csd1 and Csd2). The LytM peptidase Csd3 and the M14 peptidase Csd4 are both conserved within the Delta/Epsilonproteobacteria and Csd4 homologues showing >50% similarity to Csd4 are found only in curved and helical rod shaped organisms. The *Campylobacter jejuni* Csd4 homologue, Pgp1, also has LD-CPase activity and promotes the helical rod shape of that organism [23]. Additionally, Csd1/3-encoding Epsilonproteobacterial species with other morphologies, such as rod-shaped *Campylobacter hominis* (ATCC BAA-381) and oval-shaped *Sulfurovum* (NBC37-1), do not encode a Csd4 homologue (BLASTP *E*-values>0.1). Of the two shape-generating proteins that do not encode putative enzymes, CcmA-like bactofilins are found throughout the Proteobacteria as well as other bacterial phyla. These proteins form cytoplasmic filaments that in one case can bind a PG synthesis enzyme leading to localized activity and have been shown to cause diverse cell shape phenotypes when over or under expressed [49]–[51]. In contrast, Csd5 appears restricted to *H. pylori* and the closely related species *H. acinonychis*. Differences in the genomic carriage of these proteins may contribute to the diversity of species- and strain-specific bacterial cell shapes.

In summary, we have discovered additional components of the helical cell shape program in *H. pylori*, including a new PG modification enzyme (Csd4) and a protein (Csd5) that may localize or participate in sensing the activity of PG modification machinery. We also provided evidence that the six shape-determining proteins identified in our screen form two or more networks that cooperatively shape the cell wall through two types of cell wall modifications. For the first time we were able to establish a pattern of association between *H. pylori*'s cell shape and motility in gel-like media, bolstering the conclusion that the stomach colonization defects of *H. pylori* cell shape mutants are rooted in shape-dependent alterations of motility.

## Materials and Methods

### Ethics statement

Mouse infection studies were done under practices and procedures of Animal Biosafety Level 2 and carried out in strict accordance with the recommendations in the Guide for the Care and Use of Laboratory Animals of the National Institutes of Health. The facility is fully accredited by the Association for Assessment and Accreditation of Laboratory Animal Care and complies with all United States Department of Agriculture, Public Health Service, Washington State and local area animal welfare regulations. All activities were approved by the FHCRC Institutional Animal Care and Use Committee.

### Bacterial strains and growth

Strains used in this work, as well as primers and plasmids used in strain construction are described in Tables S3, S4 and S5 in Text S1. *H. pylori* were grown on horse blood (HB) agar plates or in Brucella broth (BD Biosciences) containing 10% fetal bovine serum (Hyclone) but no antimicrobials (BB10) under microaerobic conditions as previously described [13]. For resistance marker selection, HB plates were supplemented with 15 µg/mL chloramphenicol, 25 µg/mL kanamycin, 36 µg/mL metronidazole, or 60 mg/mL sucrose. For culturing bacteria from mouse stomachs, 200 µg/mL bacitracin was added to eliminate contaminating species of the normal mouse microbiota. For plasmid selection and maintenance in *E. coli*, LB agar or broth was supplemented with 30 µg/mL kanamycin or 100 µg/mL ampicillin.

### Phase contrast and TEM microscopy of *H. pylori* cells and quantitative morphology analyses

Phase contrast microscopy and TEM were performed as described [13]. Quantitative analysis of phase contrast images of bacteria were performed with the CellTool software package as described [13]. A detailed description of Kolmogorov–Smirnov statistical comparisons is provided in Text S1. Cell length was estimated using the central axis length calculated by CellTool for 300–350 cells/strain. Cell width was measured manually using ImageJ from TEM images (http://rsbweb.nih.gov/ij/) of 25–50 cells/strain.

### Bioinformatic analyses

Signal peptide predictions were obtained from the Comprehensive Microbial Resource web database (http://cmr.jcvi.org/tigr-scripts/CMR/CmrHomePage.cgi), structural threading was performed with Phyre [52], and 3D molecular structures were visualized using PyMOL [53]. Further detail is provided in Figure S3 in Text S1.

### PG analyses and Csd4 enzyme assay

PG was prepared from *H. pylori* cells (100–500 ODs) grown on HB plates as described [13]. Purified PG (0.5 mg/mL) was incubated with His-tagged Csd4 (5 µM) purified from *E. coli* (as described in Text S1) in 20 mM sodium phosphate, 5 mM ZnCl_2_, 100 mM NaCl, pH 4.8 for 4 hrs at 37°C on a Thermomixer at 750 rpm. A control sample received no enzyme, and another enzyme sample contained 10 mM EDTA and no ZnCl_2_. The samples were incubated with 10 µg of cellosyl (Hoechst, Frankfurt am Main, Germany) for 1 hr, boiled for 10 min and centrifuged at room temperature for 15 min at 16,000×g. The muropeptides present in the supernatant were reduced with sodium borohydride as described [54]. HPLC analysis was performed as described [13], [55]. Eluted muropeptides were detected by their absorbance at 205 nm. The muropeptide profile of the wild-type was similar to the published profile of *Helicobacter* muropeptides [18] allowing the unambiguous assignment of known muropeptide structures to the peaks detected [13]. To study the specificity of Csd4, the above assay was conducted with pure, unreduced muropeptides, the disaccharide tripeptide (0.02 mg/mL) and disaccharide tetrapeptide (0.07 mg/mL), obtained from the laboratory of J.-V. Höltje (Max-Planck-Institute, Tübingen, Germany) in lieu of PG.

### Motility, growth, and stress testing

Soft agar motility experiments were performed as described [56]. Growth and stress testing was accomplished using 100–200 µL BB10 mini-cultures grown in a 96-well plate as described [13]. For analysis of live motile cells, fresh liquid cultures were grown to an optical density of 0.5–0.7 at 600 nm (OD_600_), concentrated 10×, and kept warm at 37°C in a CO_2_ incubator. Just prior to imaging, 5–10 µL of cell concentrate was added to 100 µL of pre-warmed test solution: Brucella broth (BD Biosciences) supplemented with 5% fetal bovine serum (Hyclone, BB5), or BB5 containing 0.25–1.0% methylcellulose, 2.5–10% Ficoll PM 400, or 0.1–2.5% crude porcine mucin (all Sigma). Each cell suspension was mixed by gentle pipetting and immediately applied to a depression slide. Movies were captured using a 60× ELWD Plan Fluor (NA 0.7) objective mounted on a Nikon TE 200 microscope at a frame rate of 100 millisecond intervals with a Nikon CoolSNAP HQ CCD camera controlled by MetaMorph software (MDS Analytical Technologies). Cells were tracked using the ImageJ Manual Tracker (http://rsbweb.nih.gov/ij/) and velocity calculations performed with Intercooled Stata 8.0 (StataCorp).

### Mouse colonization experiments

Female C57BL/6 mice 24–28 days old were obtained from Charles River Laboratories and certified free of endogenous *Helicobacter* infection by the vendor. Mice were housed and infected as described [57] using 5×10^7^ cells/strain in the inocula for competition experiments. After 1 week the mice were euthanized by inhalation of CO_2_ and the glandular stomach removed and opened to remove any food. The whole stomach was homogenized in 1 mL BB10. Dilutions of homogenate were plated to non-selective and selective HB plates to enumerate bacteria of each genotype. If no bacteria were recovered we set the number of colonies on the lowest dilution plated to 1 to calculate the competitive index.

### Co-culture experiments

The human gastric adenocarcinoma cell line AGS (ATCC CRL-1739) was co-cultured with *H. pylori* strains at a multiplicity of infection of 10 for analysis of IL-8 release at 6 and 24 hrs as described previously [58].

## Supporting Information

Text S1 **Supporting information.** This file contains supplemental materials and methods, four supplemental figures, five supplemental tables, and references for the supporting information. Supplemental materials and methods describe genetic manipulations, Csd4 purification, statistical analysis of cell shape distributions, and bioinformatics analyses. Supplemental figures include Figure S1 Phylogenetic relatedness of Csd4 and Csd5 homologues and morphological complementation of their respective mutant strains, Figure S2 Growth of wild-type, csd4, and csd5 mutant strains independently and in co-culture, Figure S3 Prediction of Csd4 functional residues through structural threading analysis, and Figure S4 Morphological characterization of cross-shape class and straight rod double mutants. Supplemental tables include Table S1 Muropeptide composition of Csd4 treated Δcsd4 mutant sacculi, Table S2 Muropeptide composition of wild-type, mutant, and complemented mutant strains, Table S3 Bacterial strains, Table S4 Primers, and Table S5 Plasmids.
(PDF)

Video S1 **Video depicting motile helical wild-type and straight rod** ***csd4*** **mutant** ***H. pylori*** **in broth media.** Five second video with a frame rate of 0.1 seconds taken at 600×. The *csd4* mutant is on the left, wild-type on the right. Note that although cell morphology differences are not readily apparent at this magnification, both strains exhibit similar motility.
(MOV)

Video S2 **Video depicting motile helical wild-type and straight rod** ***csd4*** **mutant** ***H. pylori*** **in 0.5% methylcellulose.** Five second video with a frame rate of 0.1 seconds taken at 600×. The *csd4* mutant is on the left, wild-type on the right. Note that although cell morphology differences are not readily apparent at this magnification, both strains exhibit similar motility.
(MOV)
