# Supplementary material for: Multiple Peptidoglycan Modification Networks Modulate Helicobacter pylori's Cell Shape, Motility, and Colonization Potential
Source: PLoS Pathog. 2012 Mar 22;8(3):e1002603. doi: 10.1371/journal.ppat.1002603 (PMC3310797; doi:10.1371/journal.ppat.1002603)
Supplement: Text S1 — Supporting information. This file contains supplemental materials and methods, four supplemental figures, five supplemental tables, and references for the supporting information. Supplemental materials and methods describe genetic manipulations, Csd4 purification, statistical analysis of cell shape distributions, and bioinformatics analyses. Supplemental figures include Figure S1 Phylogenetic relatedness of Csd4 and Csd5 homologues and morphological complementation of their respective mutant strains, Figure S2 Growth of wild-type, csd4, and csd5 mutant strains independently and in co-culture, Figure S3 Prediction of Csd4 functional residues through structural threading analysis, and Figure S4 Morphological characterization of cross-shape class and straight rod double mutants. Supplemental tables include Table S1 Muropeptide composition of Csd4 treated Δcsd4 mutant sacculi, Table S2 Muropeptide composition of wild-type, mutant, and complemented mutant strains, Table S3 Bacterial strains, Table S4 Primers, and Table S5 Plasmids. (PDF) [file ppat.1002603.s001.pdf]

Supplemental Materials and Methods

Genetic manipulations. PCR SOEing [1] was used to create knockout alleles as described [2]. The *csd4* point mutant was generated by amplifying *csd4* with flanking primers containing XhoI and EcoRI restriction sequences and cloning the gene into a Bluescript vector (pBluescript II SK+, Invitrogen). This plasmid, pLKS2, served as the template for PCR-based site-directed mutagenesis using a QuikChange Site-Directed Mutagenesis kit (Stratagene). The presence of only the desired *csd4* point mutation (A665C) in the resulting plasmid, pLKS12, was confirmed by sequencing. Complementation at the *rdxA* locus was achieved by inserting the deleted gene into pLC292, a plasmid containing *rdxA* flanking sequences [3]. pLKS24 was generated by inserting a PCR product containing *csd4* flanked by XbaI and XhoI restriction sequences into pLC292. pLKS27 was constructed by subcloning the XbaI-SpeI fragment containing *csd5* from pLKS23, a TOPO vector containing *csd5*, into pLC292.

The first 20 amino acids of Csd4 are predicted to encode an N-terminal transmembrane domain. In order to create a Csd4 *E. coli* expression vector, we decided to clone only the portion downstream of the transmembrane domain (aa 21-438). The *csd4* gene (bp 61 through 140 bp downstream of the stop codon) was amplified by PCR using primers containing a 5' NcoI site and a 3' XhoI site. The primers were designed based on the sequence of HP1075, the *csd4* gene in *H. pylori* 26695, as the G27 sequence was not available at the time this plasmid was made. Two sequence differences relative to the G27 sequence were introduced by the forward primer: a63g, a silent mutation, and c65g, resulting in a T22M mutation with respect to the G27 amino acid sequence. The PCR product was cloned into the NcoI and XhoI sites of pET15-HE (generously provided by Barry Stoddard, FHCRC), resulting in pLKS1. This plasmid allows expression of N-terminally truncated Csd4 with an N-terminal His•Tag behind a thrombin cleavage site.

Plasmids were prepared using Qiagen kits and *H. pylori* genomic DNA isolated using the Wizard Genomic DNA kit (Promega). All restriction enzymes and high-fidelity polymerases used for PCR SOEing were obtained from New England Biolabs or Invitrogen. Sequencing was performed by the FHCRC Genomics Shared Resource and sequences analyzed using Sequencher (Gene Codes).

PCR products and plasmids were introduced into the chromosome using natural transformation as described [4] and the presence of the correct allele affirmed by PCR. Clones

were additionally checked for urease activity, flagella-based motility and the absence of undesired point mutations in the coding sequences. Single clones were used for phenotypic characterization and infection experiments. Selectable alleles were transferred between *H. pylori* strains by transforming genomic DNA using the same procedures.

Csd4 purification. BL21(DE3) cells carrying pLKS1 (encoding amino acids 21-438 of Csd4 with an N-terminal His•Tag) were grown with shaking in LB supplemented with 100 µg/mL ampicillin to mid-log phase (A_{600} 0.4-0.6). Cultures were cooled on ice for 15 minutes, induced with 1 mM IPTG, and grown overnight (16-20 hr) at 15°C with shaking. Cells were harvested by centrifugation, resuspended in 0.01 volumes cell lysis buffer (50 mM sodium phosphate pH 8, 500 mM NaCl, 10% glycerol, 10 mM $MgCl_2$, 1× Complete EDTA-free Protease Inhibitor Cocktail (Roche)), and stored at -20°C. Cells were thawed and then lysed by three additional freeze-thaw cycles (dry ice/methanol bath), followed by three sonication cycles at 10% power (10 sec total, 1 sec on/1 sec off) (Fisher Scientific Sonic Dismembrator Model 500). Cell debris was removed by centrifugation (10,000×g, 10 min).

Ni-NTA agarose (Invitrogen) was prepared by rinsing once with 5 volumes of water and then twice with 5 volumes of binding buffer (50 mM sodium phosphate pH 8, 500 mM NaCl, 10% glycerol, 10 mM $MgCl_2$). Binding was performed in batch by adding 5 volumes of cell lysate to prepared Ni-NTA agarose at 4°C for 1 hr with gentle mixing. This mixture was poured into a column and washed four times with 10 volumes of wash buffer (binding buffer plus 20 mM imidazole). Csd4 was eluted with 10 volumes of elution buffer (binding buffer plus 250 mM imidazole). Elution fractions containing approximately 95% pure Csd4 (as judged visually by Coomassie staining of SDS-PAGE) at reasonably high concentration (>0.5 mg/mL by Bradford assay) were pooled. The glycerol concentration of the pool was increased to 20% prior to storage at -20°C.

Statistical analysis of cell shape distributions. Kolmogorov–Smirnov (KS) statistics were calculated to assay the differences between the double mutant and single mutant (or wild-type) distributions of side curvature generated by CellTool. In order to determine a biologically-plausible null distribution for the range of KS statistics expected to be observed by chance due to biological variability, a bootstrapped distribution of KS values was calculated for the wild-type

and single-mutant strains as follows: the side-curvature values from each strain were resampled (with replacement) into two separate samples 10^5 times and the KS statistic computed for each pair of samples. For each comparison of a double mutant to a single mutant (or wild-type), the measured KS distance between the two distributions was compared to the null distribution calculated for the single mutant (or wild-type), generating a one-tailed p-value. As the distributions of side-curvature values are quite different for the different strains, it was most appropriate to calculate separate null distributions for each single mutant strain.

Bioinformatic analyses. Csd4/5 homologues were identified by performing a BLASTP search of the NCBI non-redundant protein sequences database using HPG27 Csd4 and Csd5 as queries. Hits with a maximum *E*-value of 0.001 were considered potential homologues, but we focused subsequent analyses on hits aligning across at least 75% of the query sequence and showing >50% similarity (determined by MatGAT 3.0 [5]). All of these homologues had BLASTP *E*-values < $1E^{-40}$. Altogether our Csd4 BLAST search yielded 108 hits with *E*-values < 0.001, 87 of which met our 50% similarity cut-off. Our Csd5 BLAST search yielded 39 hits with *E*-values < 0.001, 29 of which met our 50% similarity cut-off.

To generate the phylogenetic trees in Figure S1 we included only species/strains for which the genome was listed as "complete" by NCBI. We retained all 10 complete *H. pylori* genomes, but chose only one representative strain for the 24 other species/subspecies with Csd4 homologues. Dataset compilation and editing, multisequence alignment (through a built-in Clustal W algorithm), and construction of neighbor-joining trees were all accomplished using MEGA 5.05 [6].

Morphological classifications were drawn from multiple chapters of *The Prokaryotes* [7,8] and *Bergey's Manual of Systematic Bacteriology* [9,10]. We consulted additional primary research articles for the following species: *Arcobacter butzleri* [11,12], *Arcobacter nitrofigilis* [13], *Campylobacter concisus* and *Campylobacter curvus* [14], *Campylobacter hominis* [15], *Nautilia profundicola* [16], *Sulfuricurvum kujiense* [17], *Sulfurimonas dentrificans*[18], *Sulfurospirillum deleyianum* [19], and *Wolinella succinogenes* [20,21].

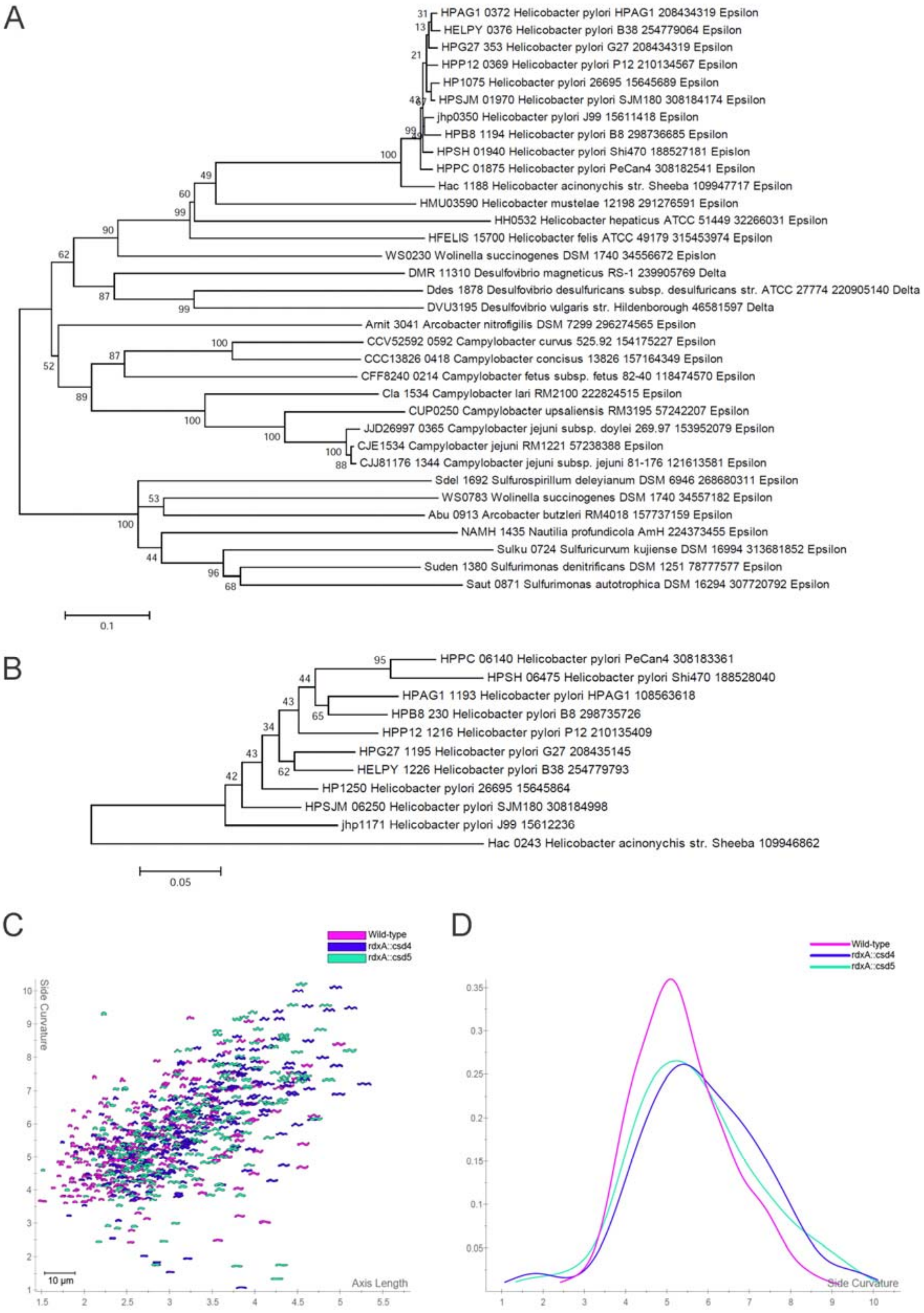
A
HPAG1 0372 Helicobacter pylori HPAG1 208434319 Epsilon
HELPY 0376 Helicobacter pylori B38 254779064 Epsilon
HPG27 353 Helicobacter pylori G27 208434319 Epsilon
HPP12 0369 Helicobacter pylori P12 210134567 Epsilon
HP1075 Helicobacter pylori 26695 15645689 Epsilon
HPSJM 01970 Helicobacter pylori SJM180 308184174 Epsilon
jhp0350 Helicobacter pylori J99 15611418 Epsilon
HPB8 1194 Helicobacter pylori B8 298736685 Epsilon
HPSH 01940 Helicobacter pylori Shi470 188527181 Epsilon
HPPC 01875 Helicobacter pylori PeCan4 308182541 Epsilon
Hac 1188 Helicobacter acinonychis str. Sheeba 109947717 Epsilon
HMU03590 Helicobacter mustelae 12198 291276591 Epsilon
HH0532 Helicobacter hepaticus ATCC 51449 32266031 Epsilon
HFELIS 15700 Helicobacter felis ATCC 49179 315453974 Epsilon
WS0230 Wolinella succinogenes DSM 1740 34556672 Epsilon
DMR 11310 Desulfovibrio magneticus RS-1 239905769 Delta
Ddes 1878 Desulfovibrio desulfuricans subsp. desulfuricans str. ATCC 27774 220905140 Delta
DVU3195 Desulfovibrio vulgaris str. Hildenborough 46581597 Delta
Arnit 3041 Arcobacter nitrofigilis DSM 7299 296274565 Epsilon
CCV52592 0592 Campylobacter curvus 525.92 154175227 Epsilon
CCC13826 0418 Campylobacter concisus 13826 157164349 Epsilon
CFF8240 0214 Campylobacter fetus subsp. fetus 82-40 118474570 Epsilon
Cla 1534 Campylobacter lari RM2100 222824515 Epsilon
CUP0250 Campylobacter upsaliensis RM3195 57242207 Epsilon
JJD26997 0365 Campylobacter jejuni subsp. doylei 269.97 153952079 Epsilon
CJE1534 Campylobacter jejuni RM1221 57238388 Epsilon
CJJ81176 1344 Campylobacter jejuni subsp. jejuni 81-176 121613581 Epsilon
Sdel 1692 Sulfurospirillum deleyianum DSM 6946 268680311 Epsilon
WS0783 Wolinella succinogenes DSM 1740 34557182 Epsilon
Abu 0913 Arcobacter butzleri RM4018 157737159 Epsilon
NAMH 1435 Nautilia profundicola AmH 224373455 Epsilon
Sulku 0724 Sulfuricurvum kujiense DSM 16994 313681852 Epsilon
Suden 1380 Sulfurimonas denitrificans DSM 1251 78777577 Epsilon
Saut 0871 Sulfurimonas autotrophica DSM 16294 307720792 Epsilon
31
13
21
100
49
60
99
90
62
87
99
52
87
100
89
100
100
100
88
53
100
44
96
68
0.1
B
HPPC 06140 Helicobacter pylori PeCan4 308183361
HPSH 06475 Helicobacter pylori Shi470 188528040
HPAG1 1193 Helicobacter pylori HPAG1 108563618
HPB8 230 Helicobacter pylori B8 298735726
HPP12 1216 Helicobacter pylori P12 210135409
HPG27 1195 Helicobacter pylori G27 208435145
HELPY 1226 Helicobacter pylori B38 254779793
HP1250 Helicobacter pylori 26695 15645864
HPSJM 06250 Helicobacter pylori SJM180 308184998
jhp1171 Helicobacter pylori J99 15612236
Hac 0243 Helicobacter acinonychis str. Sheeba 109946862
95
44
43
65
34
43
62
42
0.05
C
Wild-type
rdxA::csd4
rdxA::csd5
Side Curvature
Axis Length
10 µm
1.5
2
2.5
3
3.5
4
4.5
5
5.5
1
2
3
4
5
6
7
8
9
10
D
Wild-type
rdxA::csd4
rdxA::csd5
Side Curvature
0.35
0.3
0.25
0.2
0.15
0.1
0.05
1
2
3
4
5
6
7
8
9
10

Figure S1. Phylogenetic relatedness of Csd4 and Csd5 homologues and morphological complementation of their respective mutant strains. A-B) Dendograms displaying Csd4 (A) and Csd5 (B) homologues that show at least 50% similarity over 75% or more of the query sequence (BLASTP *E*-values < $1E^{-40}$). Sequence labels list the locus tag, species and strain name, GI number, and the Proteobacterial subdivision. Branches are labeled with bootstrapping values formulated from 1000 iterations. Homologues from all 10 complete *H. pylori* genomes are included in the analyses and cluster together. One representative strain is shown for the other 24 species/subspecies encoding Csd4 homologues. *Wolinella succinogens* is the only species that appears to encode two Csd4 homologues, but they do not cluster together suggesting they arose from an early duplication event and subsequently diverged, or that one was acquired by horizontal gene transfer. All of the species and strains shown have curved or helical rod morphology (references listed in Supporting Information Supplemental Materials and Methods) and all Csd4 homologues have conserved the catalytic glutamate that we demonstrate is required for Csd4's shape-determining function. C) Scatter plot arraying wild-type and complemented strains by length (x-axis, μm) and cell curvature (y-axis, arbitrary units). CellTool software was used to capture the polygonal outlines of 200-300 cells/strain from 1000× phase contrast images and algorithmically determine each cell's length along its two-dimensional central axis, as well as the degree of cell body curvature (excluding the poles) [2]. D) Smooth histogram displaying kernel density estimates of the distribution of each strain's cell curvature (x-axis). Strains used: NSH57, LSH38, LSH104.

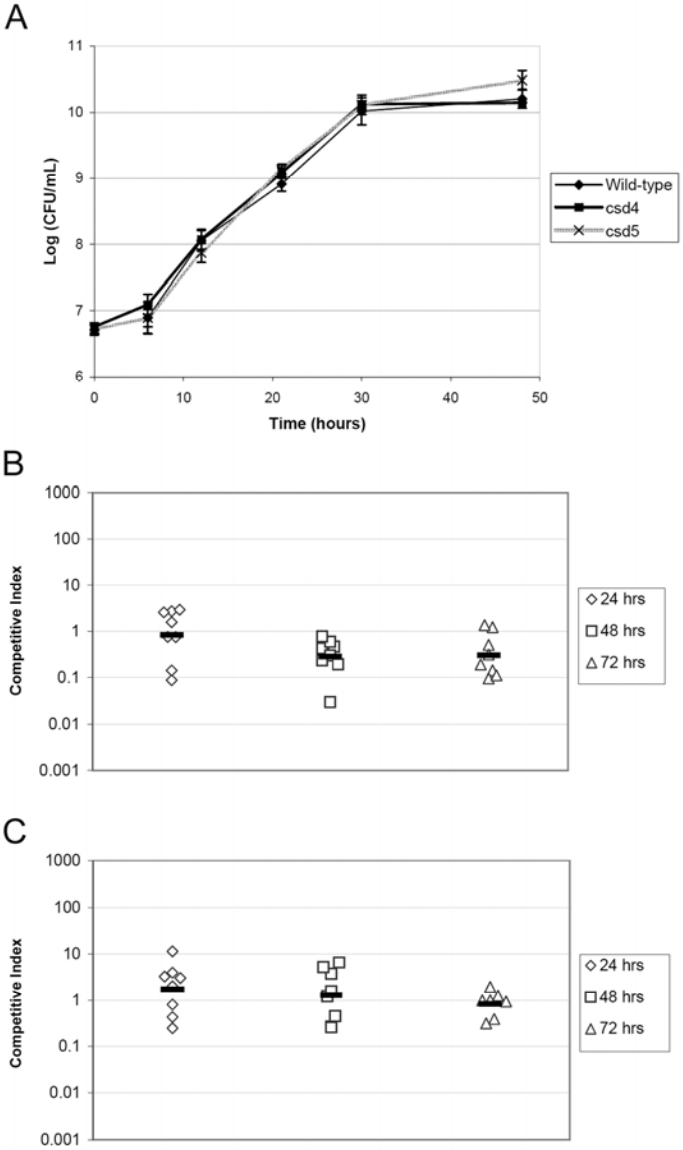
A
Log (CFU/mL)
Time (hours)
Wild-type
csd4
csd5
B
Competitive Index
24 hrs
48 hrs
72 hrs
C
Competitive Index
24 hrs
48 hrs
72 hrs

Figure S2. Growth of wild-type, *csd4,* and *csd5* mutant strains independently and in co-culture. A) Growth in liquid culture through log phase and into stationary phase. Data are compiled from two independent experiments with four replicate cultures per strain in each experiment. Data points show the mean ± SD. B,C) Results of 72 hr experiments co-culturing *csd4* (B) and *csd5* (C) mutants with wild-type. Cultures were maintained in log phase throughout the experiment by diluting every 24 hrs. Data encompass two independent experiments with four replicate cultures each, yielding a total of eight cultures (data points) for each strain pair. Data are plotted as a competitive index: [CFU/mL_{MUT}:CFU/mL_{WT} at time 24, 48, or 72 hrs]/[CFU/mL_{MUT}:CFU/mL_{WT} at time 0]. Black bars indicate the geometric mean. Strains used: NSH57, LSH18, LSH36.

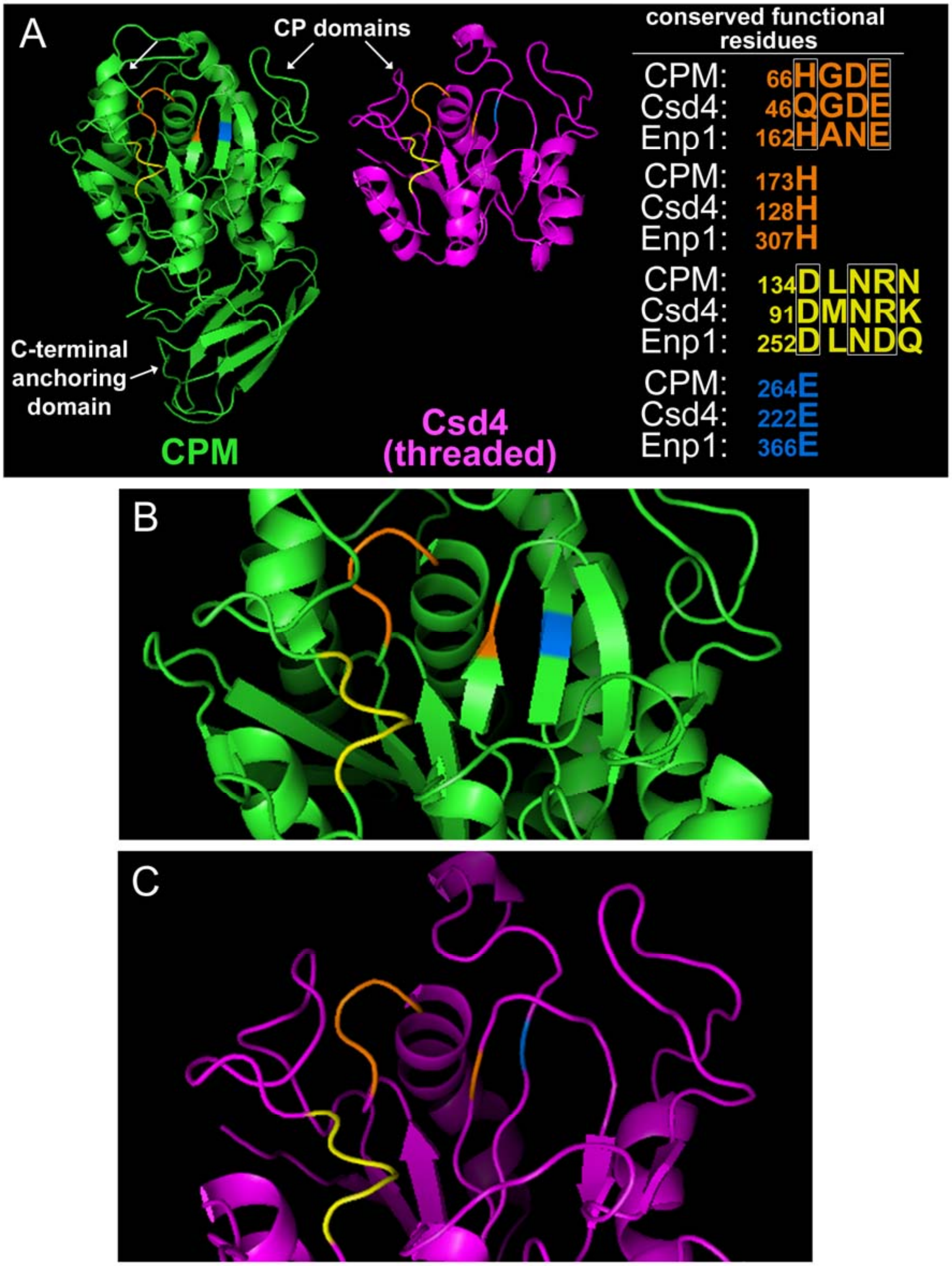
A
CP domains
conserved functional residues
CPM: 66HGDE
Csd4: 46QGDE
Enp1: 162HANE
CPM: 173H
Csd4: 128H
Enp1: 307H
CPM: 134D LNRN
Csd4: 91DMNRK
Enp1: 252D LNDQ
CPM: 264E
Csd4: 222E
Enp1: 366E
C-terminal anchoring domain
CPM
Csd4 (threaded)
B
C

Figure S3. Prediction of Csd4 functional residues through structural threading analysis. A) Human carboxypeptidase M crystal structure (CPM, green) alongside the partial threaded structure of Csd4 (purple) [22,23]. Csd4 threaded onto the catalytic carboxypeptidase (CP) domain of CPM, but lacks the C-terminal anchoring domain. The position of four residues/motifs involved in zinc binding (orange), carboxylate binding (yellow), and catalysis (blue) were identified on the CPM crystal structure and corresponding residues positionally identified on the Csd4 threaded structure. The identities of each residue/motif are listed to the right for CPM, Csd4, and *B. sphaericus* endopeptiase I (Enp1) [24]. Within each motif, the highly conserved residues are boxed in white. B-C) Enlarged view of the functionally important residues in CPM (B) and Csd4 (C), with the catalytic glutamate targeted by site-directed mutagenesis indicated in blue.

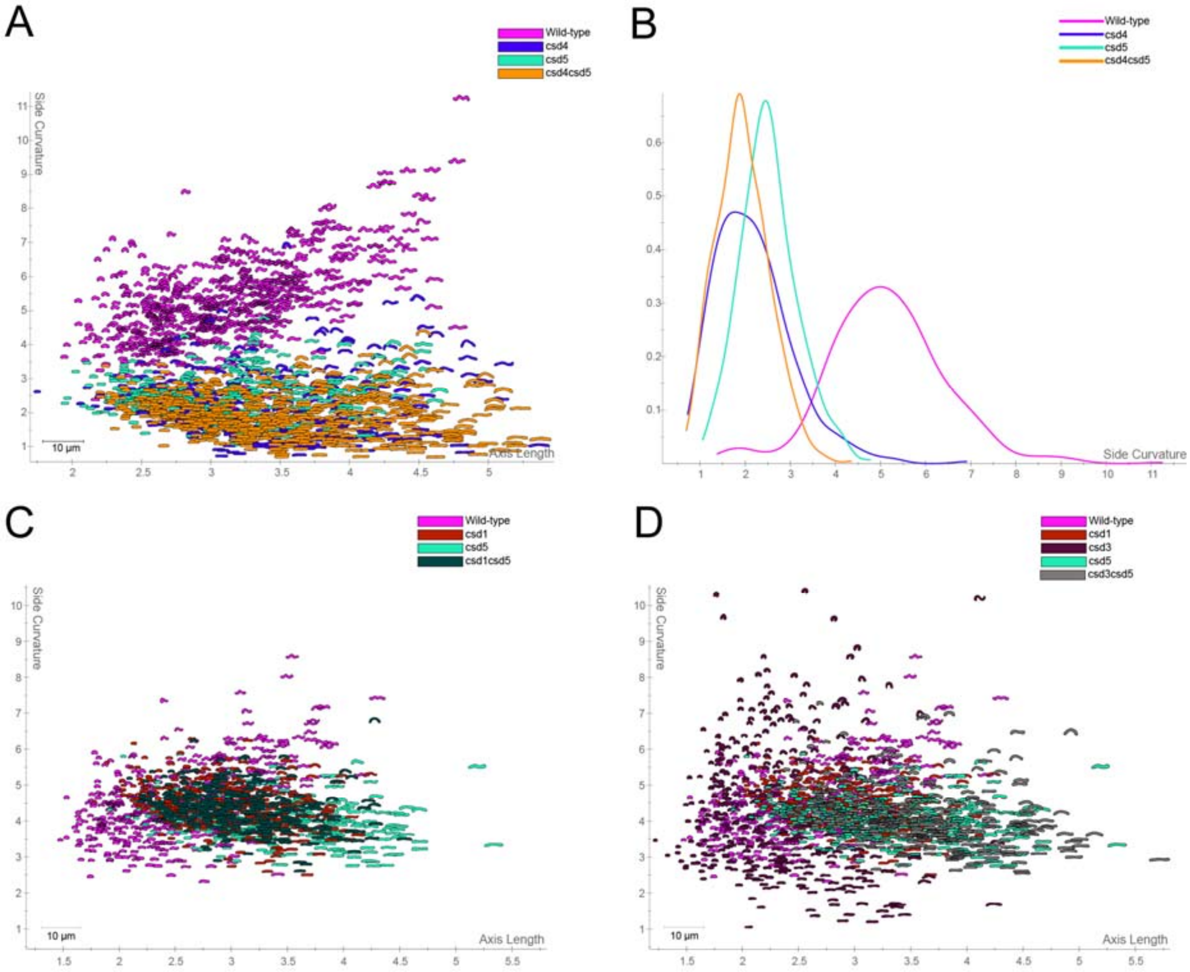
A
Wild-type
csd4
csd5
csd4csd5
Side Curvature
Axis Length
10 µm
B
Wild-type
csd4
csd5
csd4csd5
Side Curvature
C
Wild-type
csd1
csd5
csd1csd5
Side Curvature
Axis Length
10 µm
D
Wild-type
csd1
csd3
csd5
csd3csd5
Side Curvature
Axis Length
10 µm

Figure S4. Morphological characterization of cross-shape class and straight rod double mutants. A, C-D) Scatter plots arraying wild-type and mutant populations by cell length (x-axis, μm) and cell curvature (y-axis, arbitrary units). B) Smooth histograms displaying population cell curvature (x-axis) as a density function (y-axis). Bootstrapped Komolgorov–Smirnov (KS) statistics of population cell curvature distributions: A) *csd4csd5* vs. *csd4* p=0.01, *csd4csd5* vs. *csd5* p<0.00001; C) *csd1csd5* vs. *csd1* p=0.25, *csd1csd5* vs. *csd5* p<0.00001; D) *csd3csd5* vs. *csd5* p=0.37, *csd3csd5* vs. *csd3* p<0.00001, *csd3csd5* vs. *csd1* p<0.00001. All mutant comparisons to wild-type had p<0.00001. Strains used: LSH100, LSH122, LSH123, LSH134, MLH3, MLH4, NSH152a, NSH161, TSH36.

Figure S5. Video depicting motile helical wild-type and straight rod *csd4* mutant *H. pylori* in broth media. Five second video with a frame rate of 0.1 seconds taken at 600x. The *csd4* mutant is on the left, wild-type on the right. Note that although cell morphology differences are not readily apparent at this magnification, both strains exhibit similar motility.

Figure S6. Video depicting motile helical wild-type and straight rod *csd4* mutant *H. pylori* in 0.5% methylcellulose. Five second video with a frame rate of 0.1 seconds taken at 600x. The *csd4* mutant is on the left, wild-type on the right. Note that although cell morphology differences are not readily apparent at this magnification, both strains exhibit similar motility.

Table S1 Muropeptide composition of Csd4 treated *Δcsd4* mutant sacculi

		% Area[a]		
**Peak No.**	**Muropeptide**	**Buffer**	**+Csd4, EDTA**	**+Csd4, $ZnCl_2$**
1	Tri	16.52	16.68	0.20
2	Tetra	1.51	1.98	1.69
3	Penta(Gly5)	3.96	4.07	4.01
4	Di	0.00	0.00	13.29
5	Penta	30.83	31.45	31.40
6	TetraTri	7.57	6.96	7.40
7	TetraPenta(Gly5)	1.76	1.74	2.01
8	TetraTetra	3.80	3.66	3.81
9	TetraPenta	9.81	10.73	9.87
10	PentaAnh	1.45	1.23	0.82
11	TetraTriAnh I	3.17	2.40	3.19
12	TetraTriAnh II	2.61	2.06	2.61
13	TetraTetraAnh I	2.48	1.87	2.46
14	TetraTetraAnh II	1.01	0.45	0.95
15	TetraPentaAnh	6.83	5.53	6.82
1 - 15	all known	93.31	90.81	90.53

[a]Percentages calculated as per [25].

Table S2 Muropeptide composition of wild-type, mutant, and complemented mutant strains

		%Area[a]												
			**LSH 13[b]**	**LSH 112[b]**	**LSH 18**	**LSH 28**	**LSH 105**	**LSH 36**	**LSH 104**	**TSH 36**	**LSH28 -13**	**KGH 6**	**NSH 153b**	**MLH 3**
**Peak No.**	**Muropeptide**	**WT (Avg ± SD)[b]**	**csd1**	**csd3**	**csd4**	**csd4[E222A]**	**csd4 cmpl**	**csd5**	**csd5 cmpl**	**csd4 csd5**	**csd1 csd4**	**csd1 csd5**	**csd3 csd4**	**csd3 csd5**
1	Tri	3.98 ± 0.34	4.57	3.35	17.29	16.05	3.38	4.46	4.33	1.14	11.8	3.12	10.74	3.22
2	Tetra	9.59 ± 0.49	7.11	6.31	2.34	2.14	9.28	8.87	9.70	1.36	2.17	6.92	2.94	6.22
3	Penta(Gly5)	4.40 ± 0.37	5.06	5.65	4.10	3.07	4.46	5.44	3.92	3.72	5.2	5.72	5.77	6.37
4	Di	2.52 ± 0.36	1.65	2.14	0.00	0.00	2.15	2.79	2.46	0	0.36	1.79	0.00	2.2
5	Penta	33.33 ± 1.42	33.01	31.61	35.08	36.31	32.85	35.76	32.21	32.01	30.1	30.16	33.39	30.63
6	TetraTri	2.44 ± 0.24	2.7	1.97	8.40	8.61	2.25	2.90	2.64	7.88	5.09	1.94	4.20	1.76
7	TetraPenta(Gly5)	2.07 ± 0.21	2.93	3.3	1.73	1.40	2.24	2.43	1.79	2.11	3.14	3.65	3.03	3.66
8	TetraTetra	7.72 ± 0.59	8.27	5.05	4.54	4.43	8.15	8.03	7.88	3.38	5.31	7.33	2.69	4.54
9	TetraPenta	11.20 ± 1.15	16.61	16.85	11.48	12.34	12.02	12.70	11.06	10.03	13.79	14.52	14.57	16.37
10	PentaAnh	1.69 ± 0.13	1.43	1.48	2.10	2.39	1.83	1.96	1.68	1.35	1.5	1.68	1.99	1.55
11	TetraTriAnh I	1.23 ± 0.12	0.89	0.7	2.78	2.94	1.09	1.11	1.23	3.78	2.35	1.12	1.71	0.7
12	TetraTriAnh II	0.69 ± 0.08	0.24	0.51	1.64	1.65	0.58	0.72	0.81	2.3	1.19	0.41	0.96	0.46
13	TetraTetraAnh I	5.16 ± 0.59	4.69	2.95	2.52	2.37	4.06	4.74	3.92	3.32	2.69	5.85	1.90	1.83
14	TetraTetraAnh II	1.82 ± 0.24	1.02	1.17	0.73	0.70	1.64	1.57	1.90	0.8	0.87	1.19	0.68	1.15
15	TetraPentaAnh	7.06 ± 0.29	6.44	9.15	5.28	5.61	7.13	6.53	7.38	7.27	6.88	7.43	7.47	8.59
1-15	all known	94.92 ± 2.74	96.63	92.18	100	100	93.11	100	92.91	96.45	92.44	92.83	92.04	89.25

[a]Percentages calculated as per [25].
[b]As reported in [2].
[c]Calculated from 6 independent samples.

Table S3 Bacterial strains

Name	Relevant Genotype or Description	Reference or Source
G27	Wild-type *H. pylori*	[26]
NSH57	Wild-type *H. pylori*	[27]
LSH100	Wild-type *H. pylori*, NSH57 with G27 *fliM* allele	[28]
P8D1#51	*csd4::tn-cat* in G27	This study
LSH13	Δ*csd1::catsacB* in NSH57	[2]
LSH16	Δ*csd4::cat* in NSH57	This study
LSH18	Δ*csd4::catsacB* in NSH57	This study
LSH28	$csd4^{A665C}$ (or *csd4E222A*) in NSH57	This study
LSH28_13	*csd4E222A* Δ*csd1::catsacB* in NSH57	This study
LSH31	Δ*csd5::catsacB* in NSH57	This study
LSH36	Δ*csd5::catsacB* in NSH57 (back-cross)	This study
LSH45	*rdxA::catsacB** (sacB gene inactivated) in NSH57	This study
LSH80	Δ*csd4::cat rdxA::csd4* in NSH57	This study
LSH91	Δ*csd5::cat* in NSH57	This study
LSH104	Δ*csd5::cat rdxA::csd5* in NSH57	This study
LSH105	Δ*csd4::cat rdxA::csd4* in NSH57	This study
LSH122	Δ*csd4::cat* in LSH100	This study
LSH123	Δ*csd5::cat* in LSH100	This study
LSH124	Δ*csd4::cat rdxA::csd4* in LSH100	This study
LSH134	Δ*csd1::aphA3* in LSH100	[2]
KGH2	Δ*csd1::aphA3* in NSH57	This study
KGH6	Δ*csd5::cat* Δ*csd1::aphA3* in NSH57	This study
MLH3	Δ*csd5::cat* Δ*csd3::aphA3* in LSH100	This study
MLH4	Δ*csd5::cat* Δ*csd3::aphA3* in LSH100	This study
NSH152a	Δ*csd3::aphA3* in LSH100	This study
NSH153a	Δ*csd3::aphA3 csd4E222A* in LSH100	This study
NSH153b	Δ*csd3::aphA3 csd4E222A* in LSH100	This study
NSH160a	Δ*csd1::aphA3 csd4E222A* in LSH100	This study
NSH161	Δ*csd1::aphA3 csd5::cat* in LSH100	This study
TSH36	Δ*csd4::aphA3sacB csd5::cat* in LSH100	This study
XL-1 Blue	Cloning *E. coli* strain	Stratagene
Top10	Cloning *E. coli* strain	Invitrogen
DH10B	Cloning *E. coli* strain	Invitrogen
BL21(DE3)	Protein expression *E. coli* strain	Invitrogen

Table S4 Primers

Name	Sequence
Mapping transposon insertions	
N3	TTTAATACGACGGGCAATTTGCACTTCAG
N2	CAGTTTAAGACTTTATTGTC
S	TAATCCTTAAAAACTCCATTTCCACCCCT
S2	AGTTCCCAACTATTTTGTCC
CEKG2A	GGCCACGCGTCGACTAGTACNNNNNNNNNNAGAG
CEKG2C	GGCCACGCGTCGACTAGTACNNNNNNNNNNGATAT
CEKG4	GCCACGCGTCGACTAGTAC
Generation of knockout alleles	
HPG27_353-359u-5-XhoI	CCCTCGAGATGATAGAAGCTTGCAAAGCG
HPG27_353-47-C1-3	ATCCACTTTTCAATCTATATCAACACCCAAGAATACAAG CCC
HPG27_353-1279-SB2-5	CAAAAGAAAATGCCGATATCCAACGCGTTTAGCGGGAT GATC
HPG27_353-1279-C2-5	CCCAGTTTGTCGCACTGATAAAACGCGTTTAGCGGGAT GATC
HPG27_353-356d-3-EcoRI	TAGAATTCAACATGCATAGCTCCATCAGG
HPG27_1195-256u-5	TGCGATTTTATTAGTGGTGGC
HPG27_1195-63-C1-3	ATCCACTTTTCAATCTATATCCGCTAAAACCACCAACAA AGG
HPG27_1195-529-SB2-5	CAAAAGAAAATGCCGATATCCCACGAAACAAAGGGCTA TGTG
HPG27_1195-490-C2-5	CCCAGTTTGTCGCACTGATAAAGCGTGAAGGTTTTAGA AATCC
HPG27_1195-315d-3	CCCTAAAGCGTCGGTATTGTA
HPG27_1481-389u-5	GCGAGCGCTGATGGGATTGTG
HPG27_1481-16-K2-3	GAATTGTTTTAGTACCTAGATGTCCGTAACCATTACAAT CAAACG
HPG27_1481-16-C1-3	ATCCACTTTTCAATCTATATCGTCCGTAACCATTAC AATCAAACT
HPG27_1481-789-K1-5	CGCTTCATAGAGTTATTCTGTCCCCATGAGTTTCACCA AATGG
HPG27_1481-789-SB2-5	CAAAAGAAAATGCCGATATCCCCCCATGAGTTTCAC CAAATGG
HPG27_1481-253d-3	ATATTCTTAGGGCGAGTCTCCC
Site-directed Mutagenesis	
HPG27_353-E222A-sense	GAGCGCTTTTGCCAATGCAGCCAGCAAAGAACTCC
HPG27_353-E222A-antisense	GGAGTTCTTTGCTGGCTGCATTGGCAAAAGCGCTC
Complementation	
HPG27_353-359u-5-XbaI	CCTCTAGAATGATAGAAGCTTGCAAAGCG
HPG27_353-128d-3-XhoI	ATCTCGAGGCATAGCCTCTCTGTTAAGTC

Expression Vector Cloning	
HPG27_353-61-5-NcoI	TCCCATGGAGATGATAGAAAAAGCCCTG

Other Sequencing	
HPG27_353-535-3	GGGGTGCAATAAATGGGCGTT
HPG27_353-422-5	CGATGCTCAATCCTAAGCGCT
HPG27_353-910-5	ATCCCCATAGAGAGCAACGCT
HPG27_353-995-3	GTCATGAGCTTGTTGCCGTAT
HPG27_353-1279-5	AACGCGTTTAGCGGGATGATC
HPG27_353-47-3	AACACCCAAGAATACAAGCCC
HPG27_1195-63-3	CGCTAAAACCACTAATGATGG
HPG27_1195-116-RT-5	AAGACAGCGCTCCAATAAGCC
HPG27_1195-270-RT-3	TGCGGTCGTTTCTAAAGGTGGC
sac-1380-seq-5	AGACAGCATCCTTGAACAAGG
cat-87-seq-3	CCTCCGTAAATTCCGATTTGT

Table S5 Plasmids

Name	Relevant Genotype	Reference or Source
pBluescript II SK+	Bluescript cloning vector	Invitrogen
pCR 2.1-TOPO	TOPO cloning vector	Invitrogen
pET15-HE	Modified pET15 expression vector	Barry Stoddard lab, FHCRC
pLC292	pRdxA with ampicillin resistance marker	[3]
pLKS1	*csd46His* in pET15HE	This study
pLKS2	*csd4* in pBluescript II SK+	This study
pLKS12	*csd4*^{A665C} generated in pLKS2	This study
pLKS23	*csd5* in pCR 2.1-TOPO	This study
pLKS24	*csd4* in pLC292	This study
pLKS27	*csd5* in pLC292	This study

References for Supporting Information

1. Horton RM (1995) PCR-mediated recombination and mutagenesis. SOEing together tailor-made genes. Mol Biotechnol 3: 93-99.
2. Sycuro LK, Pincus Z, Gutierrez KD, Biboy J, Stern CA, et al. (2010) Peptidoglycan crosslinking relaxation promotes *Helicobacter pylori's* helical shape and stomach colonization. Cell 141: 822-833.
3. Terry K, Williams SM, Connolly L, Ottemann KM (2005) Chemotaxis plays multiple roles during *Helicobacter pylori* animal infection. Infect Immun 73: 803-811.
4. Humbert O, Salama NR (2008) The *Helicobacter pylori* HpyAXII restriction-modification system limits exogenous DNA uptake by targeting GTAC sites but shows asymmetric conservation of the DNA methyltransferase and restriction endonuclease components. Nucleic Acids Res 36: 6893-6906.
5. Campanella JJ, Bitincka L, Smalley J (2003) MatGAT: an application that generates similarity/identity matrices using protein or DNA sequences. BMC Bioinformatics 4: 29.
6. Tamura K, Peterson D, Peterson N, Stecher G, Nei M, et al. (2011) MEGA5: Molecular Evolutionary Genetics Analysis using Maximum Likelihood, Evolutionary Distance, and Maximum Parsimony Methods. Mol Biol Evol.
7. Solnick JV, O'Rourke JL, Vandamme P, Lee A (2006) The Genus *Helicobacter*. In: Martin (Editor-in Chief) D, Falkow S, Rosenberg E, Schleifer KH, Stackebrandt E, editors. The Prokaryotes. 3rd ed. New York: Springer. pp. 139-177.
8. Wassenaar TM, Newell DG (2006) The Genus *Campylobacter*. In: Martin (Editor-in Chief) D, Falkow S, Rosenberg E, Schleifer KH, Stackebrandt E, editors. The Prokaryotes. 3rd ed. New York: Springer. pp. 119-138.
9. Kuever J, Rainey FA, Widdel F (2005) Family I. *Desulfovibrionaceae* fam. nov. In: Brenner DJ, Krieg NR, Staley JT, Garrity GM, editors. Bergey's Manual. 2nd edn ed. New York: Springer. pp. 926.
10. Vandamme P, Dewhirst FE, Paster BJ, On SLW (2005) Genus I. *Campylobacter*. In: Garrity (Editor-in Chief) GM, Brenner DJ, Krieg NR, Staley JT, editors. Bergey's Manual of Systematic Bacteriology 2nd ed. New York: Springer. pp. 1147-1160.
11. Kiehlbauch JA, Brenner DJ, Nicholson MA, Baker CN, Patton CM, et al. (1991) *Campylobacter butzleri sp. nov.* isolated from humans and animals with diarrheal illness. J Clin Microbiol 29: 376-385.
12. Miller WG, Parker CT, Rubenfield M, Mendz GL, Wosten MM, et al. (2007) The complete genome sequence and analysis of the epsilonproteobacterium *Arcobacter butzleri*. PLoS One 2: e1358.
13. Pati A, Gronow S, Lapidus A, Copeland A, Glavina Del Rio T, et al. Complete genome sequence of *Arcobacter nitrofigilis* type strain (CI). Stand Genomic Sci 2: 300-308.
14. Gunther IV NW, Chen CY (2009) The biofilm forming potential of bacterial species in the genus *Campylobacter*. Food Microbiol 26: 44-51.
15. Lawson AJ, On SL, Logan JM, Stanley J (2001) *Campylobacter hominis sp. nov.*, from the human gastrointestinal tract. Int J Syst Evol Microbiol 51: 651-660.
16. Campbell BJ, Jeanthon C, Kostka JE, Luther GW, 3rd, Cary SC (2001) Growth and phylogenetic properties of novel bacteria belonging to the epsilon subdivision of the Proteobacteria enriched from *Alvinella pompejana* and deep-sea hydrothermal vents. Appl Environ Microbiol 67: 4566-4572.

17. Kodama Y, Watanabe K (2004) *Sulfuricurvum kujiense gen. nov., sp. nov.*, a facultatively anaerobic, chemolithoautotrophic, sulfur-oxidizing bacterium isolated from an underground crude-oil storage cavity. Int J Syst Evol Microbiol 54: 2297-2300.
18. Takai K, Suzuki M, Nakagawa S, Miyazaki M, Suzuki Y, et al. (2006) *Sulfurimonas paralvinellae sp. nov.*, a novel mesophilic, hydrogen- and sulfur-oxidizing chemolithoautotroph within the Epsilonproteobacteria isolated from a deep-sea hydrothermal vent polychaete nest, reclassification of *Thiomicrospira denitrificans* as S*ulfurimonas denitrificans comb. nov.* and emended description of the genus *Sulfurimonas*. Int J Syst Evol Microbiol 56: 1725-1733.
19. Sikorski J, Lapidus A, Copeland A, Glavina Del Rio T, Nolan M, et al. (2010) Complete genome sequence of *Sulfurospirillum deleyianum* type strain (5175). Stand Genomic Sci 2: 149-157.
20. Baar C, Eppinger M, Raddatz G, Simon J, Lanz C, et al. (2003) Complete genome sequence and analysis of *Wolinella succinogenes*. Proc Natl Acad Sci U S A 100: 11690-11695.
21. Wolin MJ, Wolin EA, Jacobs NJ (1961) Cytochrome-producing anaerobic *Vibrio succino*genes, sp. n. J Bacteriol 81: 911-917.
22. Kelley LA, Sternberg MJ (2009) Protein structure prediction on the Web: a case study using the Phyre server. Nat Protoc 4: 363-371.
23. Reverter D, Maskos K, Tan F, Skidgel RA, Bode W (2004) Crystal structure of human carboxypeptidase M, a membrane-bound enzyme that regulates peptide hormone activity. J Mol Biol 338: 257-269.
24. Makarova KS, Grishin NV (1999) The Zn-peptidase superfamily: functional convergence after evolutionary divergence. J Mol Biol 292: 11-17.
25. Glauner B (1988) Separation and quantification of muropeptides with high-performance liquid chromatography. Anal Biochem 172: 451-464.
26. Covacci A, Censini S, Bugnoli M, Petracca R, Burroni D, et al. (1993) Molecular characterization of the 128-kDa immunodominant antigen of *Helicobacter pylori* associated with cytotoxicity and duodenal ulcer. Proc Natl Acad Sci U S A 90: 5791-5795.
27. Baldwin DN, Shepherd B, Kraemer P, Hall MK, Sycuro LK, et al. (2007) Identification of *Helicobacter pylori* genes that contribute to stomach colonization. Infect Immun 75: 1005-1016.
28. Lowenthal AC, Hill M, Sycuro LK, Mehmood K, Salama NR, et al. (2009) Functional Analysis of the *Helicobacter pylori* Flagellar Switch Proteins. J Bacteriol 191: 7147-7156.
